# Modulation of the Selectivity in Anions Recognition Processes by Combining Hydrogen- and Halogen-Bonding Interactions

**DOI:** 10.3390/molecules22122273

**Published:** 2017-12-20

**Authors:** Fabiola Zapata, Sergio J. Benítez-Benítez, Paula Sabater, Antonio Caballero, Pedro Molina

**Affiliations:** Dto de Química Orgánica, Universidad de Murcia, Campus de Espinardo, E-30100 Murcia, Spain; fazafer@um.es (F.Z.); sergiojose.benitez@um.es (S.J.B.-B.); paula.sabater@um.es (P.S.)

**Keywords:** halogen-bonding, hydrogen-bonding, anion, fluorescence, imidazolium, recognition, sensing

## Abstract

Most of the halogen bonding receptors for anions described use halogen bonding binding sites solely in the anion recognition process; only a few examples report the study of anion receptors in which the halogen bonding interaction has been used in combination with any other non-covalent interaction. With the aims to extend the knowledge in the behaviour of this kind of mixed receptors, we report here the synthesis and the anion recognition and sensing properties of a new halogen- and hydrogen- bonding receptor which binds anions by the cooperation of both non-covalent interactions. Fluorescence studies showed that the behaviour observed in the anion recognition sensing is similar to the one previously described for the halogen analogue and is quite different to the hydrogen one. On the other hand, the association constants obtained by ^1^H-NMR data demonstrate that the mixed halogen- and hydrogen-bonding receptor is more selective for SO_4_^2−^ anion than the halogen or hydrogen analogues.

## 1. Introduction

The field of anion recognition chemistry has become one of the most important areas in supramolecular chemistry due to the important role that anions play in numerous biological and environmental processes [1,2,3,4].

The hydrogen bond (HB) has been probably the most non-covalent interaction used in the design of anion receptors in the last two decades in which amides [5], ureas [6], pyrroles [7] or imidazole [8,9] among others have been uses as hydrogen donors as anion binding sites.

The use of the halogen bonding (XB) interaction as an alternative to the hydrogen bonding in the design of new anion receptors has emerged strongly in the last years. Halogen bonding is a noncovalent bonding interaction between halogen atoms that function as electrophilic centers (Lewis acids) and neutral or anionic Lewis bases [10]. Theoretical calculations indicate that the origin of this attraction arises from the positive electrostatic potential located at the terminus of the R–X axis (σ hole), thus resulting in a strongly linear geometry that maximizes the interface of opposite charges [11]. Although a number of solid state examples and theoretical studies revealed the potential of halogen bonding interactions for anion recognition, only very recently has it been exploited in solution phase. Practically all the halogen bonding receptors for anion described in the literature use halogen bonding binding sites solely to bind anions. Despite that the combination of two or more different non-covalent interactions in the same receptors is becoming an emerging strategy in the design of new anion receptors [12], only a few examples use the halogen bonding interaction in combination with others in the anion recognition process [13,14,15,16,17,18,19,20,21,22,23].

**Figure 1:**
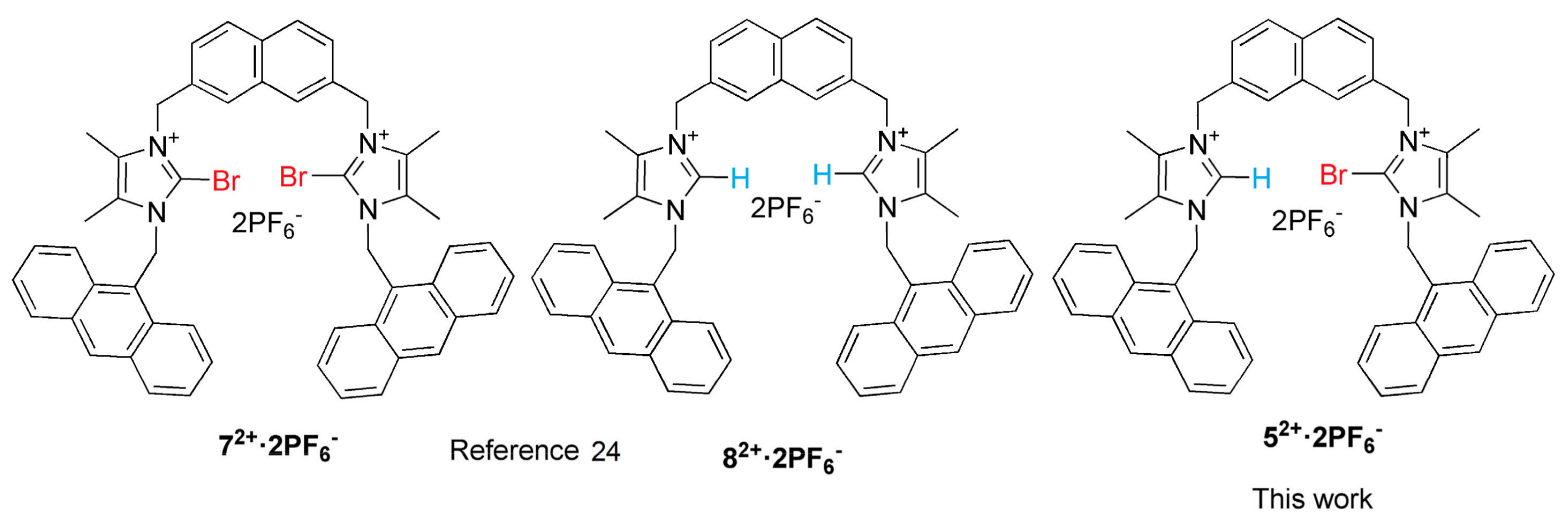
Structure of the halogen and hydrogen bonding receptors **7^2+^·2PF_6_^−^** and **8^2+^·2PF_6_^−^** reported in [24] and the structure of the mixed halogen and hydrogen bonding receptor **5^2+^·2PF_6_^−^** proposed in this work.

Recently, we have reported the synthesis and the anion sensing properties of two-armed charge-assisted ditopic imidazolium and haloimidazolium bidentade receptors which recognize anions by hydrogen or halogen bonding interaction, respectively [24]. Taking into account the important differences found in the sensing behavior between these two receptors, we decided to perform the synthesis and study of a new charge-assisted bidentade receptor containing both halogen and hydrogen bonding sites by incorporation in its structure of one haloimidazolium, halogen bonding binding site, and one imidazolium, hydrogen bonding binding site, which could act through the cooperative and simultaneous action of halogen bonds and hydrogen bonds during the anion recognition event. The receptor also incorporates two end-caped photoactive anthracene rings as fluorescent signaling units into this host framework (Figure 1).

## 2. Results and Discussion

### 2.1. Synthesis

**Scheme 1:**
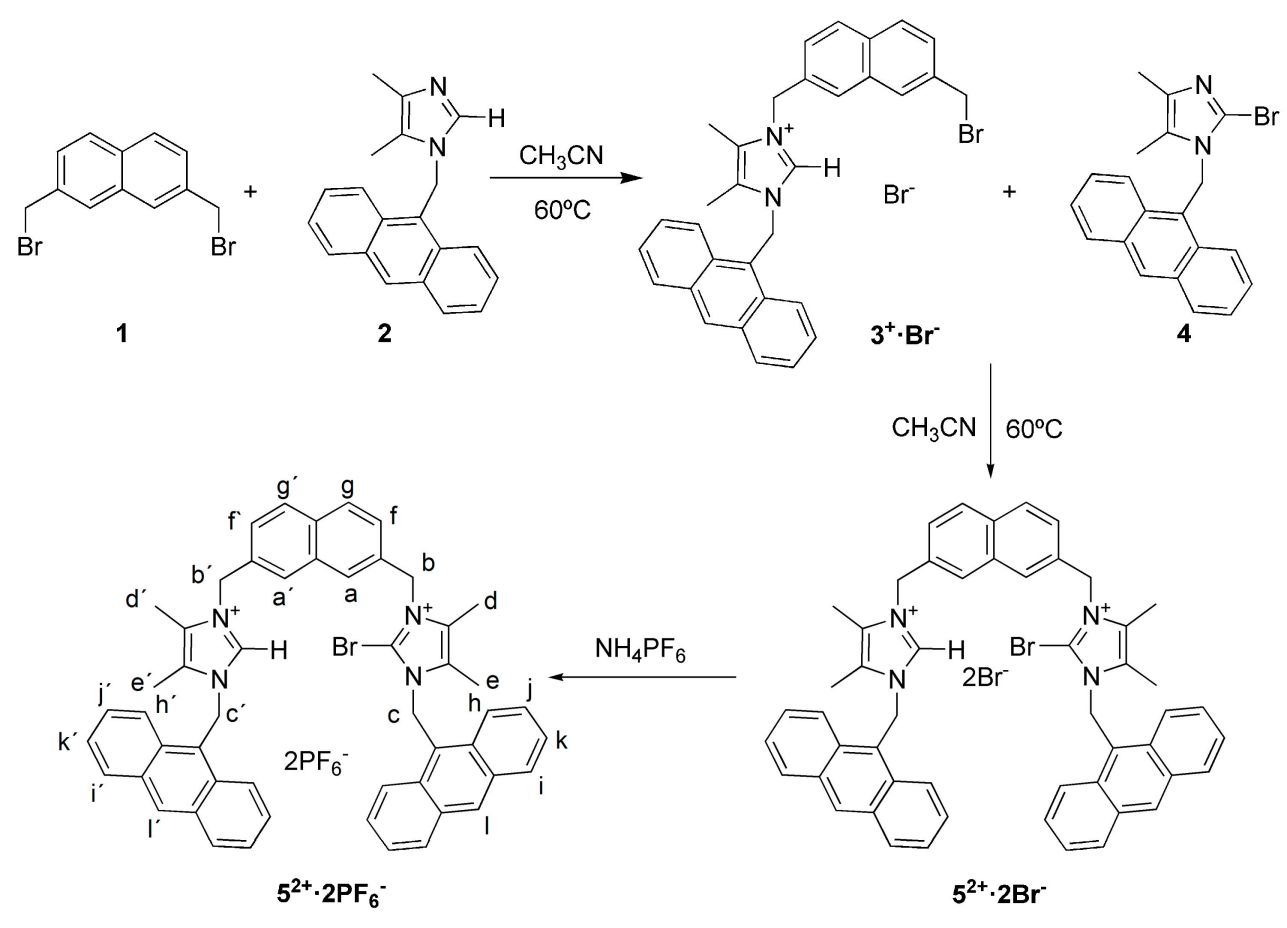
Synthesis of the halogen and hydrogen bonding receptor **5^2+^·2PF_6_^−^**.

The novel bis-imidazolium receptor **5^2+^·2PF_6_^−^** was prepared with a 31% overall yield by a stepwise procedure that involved the alkylation of 1-((anthracen-9-yl)methyl)-4,5-dimethyl-1*H*-imidazole **2** with 2,7-bis(bromomethyl)naphthalene **1**, providing the intermediate **3^+^·Br^−^** (63% yield), which was reacted with 1-((anthracen-9-yl)methyl)-2-bromo-4,5-dimethyl-1*H*-imidazole **4**, yielding the receptor as bromide salt in moderate yield (25%). Anion exchange with the corresponding hexafluorophosphate salt was achieved on addition of aqueous NH_4_PF_6_. The compounds **2** and **4** were synthesized by alkylation of 4,5-dimethyl-1*H*-imidazole [25] and 2-bromo-4,5-dimetil-1*H*-imidazol [26] with 9-(bromomethyl)anthracene, respectively (Scheme 1).

### 2.2. Anion Binding Studies

The binding and sensing ability of the receptor **5^2+^·2PF_6_^−^** toward several anions (F^−^, Cl^−^, Br^−^, I^−^, SO_4_^2−^, HSO_4_^−^, PhCOO^−^, ClO_4_^−^, HP_2_O_7_^3−^, AcO^−^, NO_3_^−^ and H_2_PO_4_^−^) as tetrabutylammonium salts were investigated by spectroscopic measurements and ^1^H-NMR spectroscopy.

**Figure 2:**
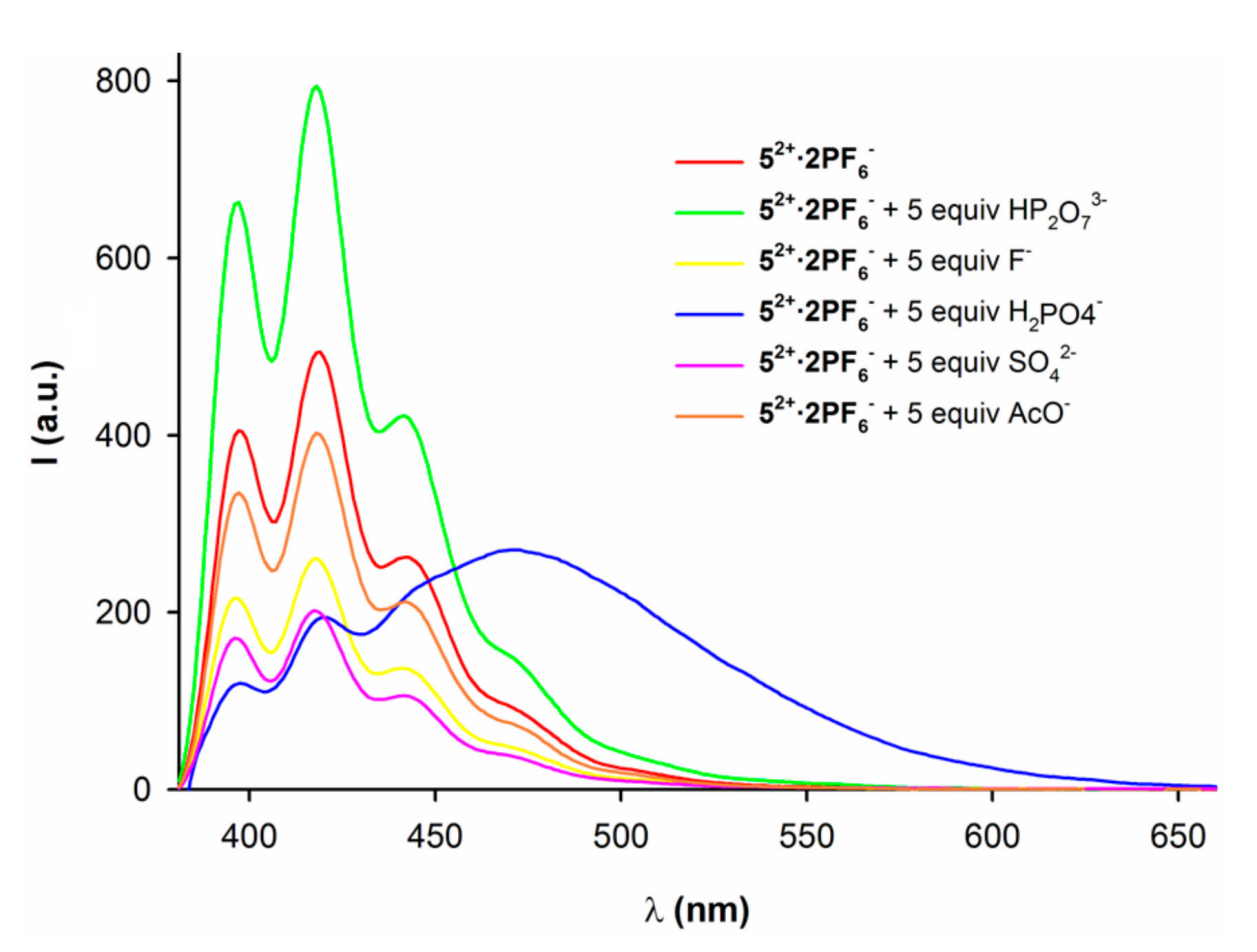
Emission spectrum of the receptor **5^2+^·2PF_6_^−^** (*c* = 1 × 10^−5^ M) in CH_3_CN (red line) and after the addition of 5 equiv. of H_2_PO_4_^−^ (blue line), SO_4_^2−^ (pink line), AcO^−^ (orange line), HP_2_O_7_^3−^ (green line) and F^−^ (yellow line).

The emission spectrum of the receptor **5^2+^·2PF_6_^−^** (Φ = 0.036) in CH_3_CN showed the characteristic anthracene monomeric bands at λ = 396, 418 and 442 nm, when excited at λ = 370 nm. The addition of the above-mentioned set of anions to a solution of the receptor **5^2+^·2PF_6_^−^** in CH_3_CN (*c* = 1 × 10^−5^ M) showed that Cl^−^, Br^−^, I^−^, HSO_4_^−^, PhCOO^−^, ClO_4_^−^ and NO_3_^−^ anions had no effect on the emission spectrum, whereas the addition of F^−^, HP_2_O_7_^3−^, SO_4_^2−^, AcO^−^ and H_2_PO_4_^−^ anions promoted significant changes in the emission spectrum of receptor **5^2+^·2PF_6_^−^**. These changes were strongly dependent on the anion added and different responses have been observed (Figure 2).

**Figure 3:**
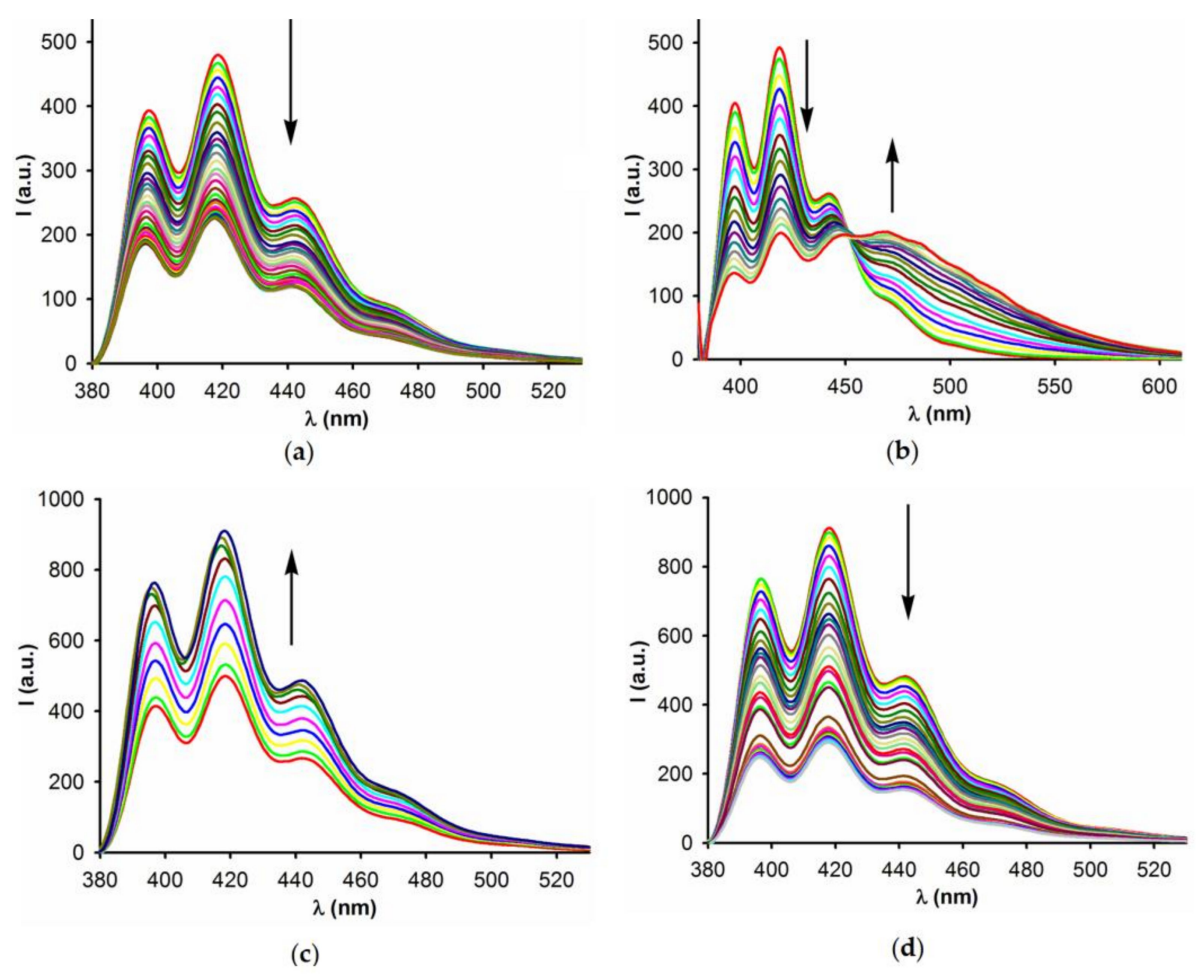
Changes in the fluorescence spectra of receptor **5^2+^·2PF_6_^−^** (*c* = 1 × 10^−5^ M) in CH_3_CN upon addition of (**a**)SO_4_^2−^, (**b**) H_2_PO_4_^−^, (**c**) HP_2_O_7_^3−^ from 0 to 1.8 equiv., and (**d**) HP_2_O_7_^3−^ from 1.8 to 100 equiv. Arrows indicate the emission bands that increase or decrease during the titration.

The presence of AcO^−^ (Figure S3, Supplementary Material) and SO_4_^2−^ anions (Figure 3a) induced a decrease in the monomer emission band at λ = 419 nm of the receptor **5^2+^·2PF_6_^−^** I_receptor_/I_complex_ = 2.55 and 2.13, respectively, and also a decrease of the quantum yield from Φ = 0.036 to Φ = 0.025 for AcO^−^ and Φ = 0.019 for SO_4_^2−^ anions. A different fluorescent response was observed for H_2_PO_4_^−^ anions (Figure 3b) in which the presence of this anion promoted a remarkable decrease of the monomer emission bands at λ = 396, 418 and 412 nm and a progressive increase in a new broad and structureless emission band at λ = 465 nm, attributed to the anthracene excimer emission band (Φ = 0.058) and finally, when HP_2_O_7_^3−^ (Figure 3c) and F^−^ (Figures S1 and S2) anions were added, two different effects were observed: first the addition up to 1.8 equiv. of HP_2_O_7_^3−^ anions or 1 equiv. F^−^ anion induced an increase in the monomer emission band of the receptor, subsequent addition of more than 1.8 equiv. of HP_2_O_7_^3−^ anions and 1 equiv. in the case of F^−^, promoted a continuous decrease in the monomer emission band.

A comparative analysis of the response observed in the fluorescence study indicates that the mixed hydrogen- and halogen- bonding receptor **5^2+^·2PF_6_^−^** shows changes similar to the halogen bonding receptor **7^2+^·2PF_6_^−^** Both receptors **5^2+^·2PF_6_^−^** (HB and XB) and **7^2+^·2PF_6_^−^** (XB) act through a photoinduced electron transfer (PET) mechanism in the presence of SO_4_^2−^ anions, and therefore a decrease in the intensity of the emission bands was observed [27]. In addition, a broad emission band at λ = 465 nm due to a π-stacking interaction between the two anthracene units [28] appears in the spectrum of the receptor **5^2+^·2PF_6_^−^** during the addition of H_2_PO_4_^−^ anions as it was also observed in the halogen bonding receptor **7^2+^·2PF_6_^−^**. Interestingly, the remarkable changes observed in the emission spectrum of the receptor **5^2+^·2PF_6_^−^** after addition of HP_2_O_7_^3−^ and F^−^ anions are consistent with a chemodosimeter behavior, which was also observed in the halogen bonding receptor **7^2+^·2PF_6_^−^**. Interestingly, the emission spectrum of the receptor **5^2+^·2PF_6_^−^** undergoes important changes in the presence of AcO^−^ anions to difference with the described for the halogen bonding receptor **7^2+^·2PF_6_^−^**, whereas the behavior towards H_2_PO_4_^−^ and SO_4_^2−^ anions is similar to that found for **7^2+^·2PF_6_^−^**. On the other hand, the fluorescent response of the mixed XB and HB receptor **5^2+^·2PF_6_^−^** is quite different than for the hydrogen bonding receptor **8^2+^·2PF_6_^−^**, in which the receptor acted on by a photoinduced electron transfer mechanism with all the tested anions.

The stoichiometry of the complexes was determinate by Job-plot experiments using the changes in the fluorogenic response of the receptor **5^2+^·2PF_6_^−^** in the presence of varying concentrations of the tested anions. The results clearly indicated the formation of 1:2 complexes in the case of H_2_PO_4_^−^ (Figure S7) and AcO^−^ anions (Figure S9), and 1:1 for SO_4_^2−^ anions (Figure S8). The association constant values were calculated by fitting the fluorescence data in CH_3_CN with the host-guest binding model obtained using the Dynafit program [29] and are summarized in Table 1 together with the values previously reported for the halogen bonding receptor **7^2+^·2PF_6_^−^** and the hydrogen bonding receptor **8^2+^·2PF_6_^−^**. The association constants obtained indicate that the hydrogen bonding receptor **8^2+^·2PF_6_^−^** binds the recognized anions stronger than the analogous halogen **7^2+^·2PF_6_^−^** or the mixed halogen and hydrogen bonding receptor **5^2+^·2PF_6_^−^** in the pure organic solvent CH_3_CN, while the monohalogenated receptor **5^2+^·2PF_6_^−^** and the dihalogenated receptor **7^2+^·2PF_6_^−^** bind the anions with similar strength.

The calculated detection limits of the receptor **5^2+^·2PF_6_^−^** for H_2_PO_4_^−^, SO_4_^2−^, and AcO^−^ anions were 3.90 × 10^−6^ M, 3.57 × 10^−5^ M, and 4.59 × 10^−5^ M respectively (Figures S4–S6).

**Figure 4:**
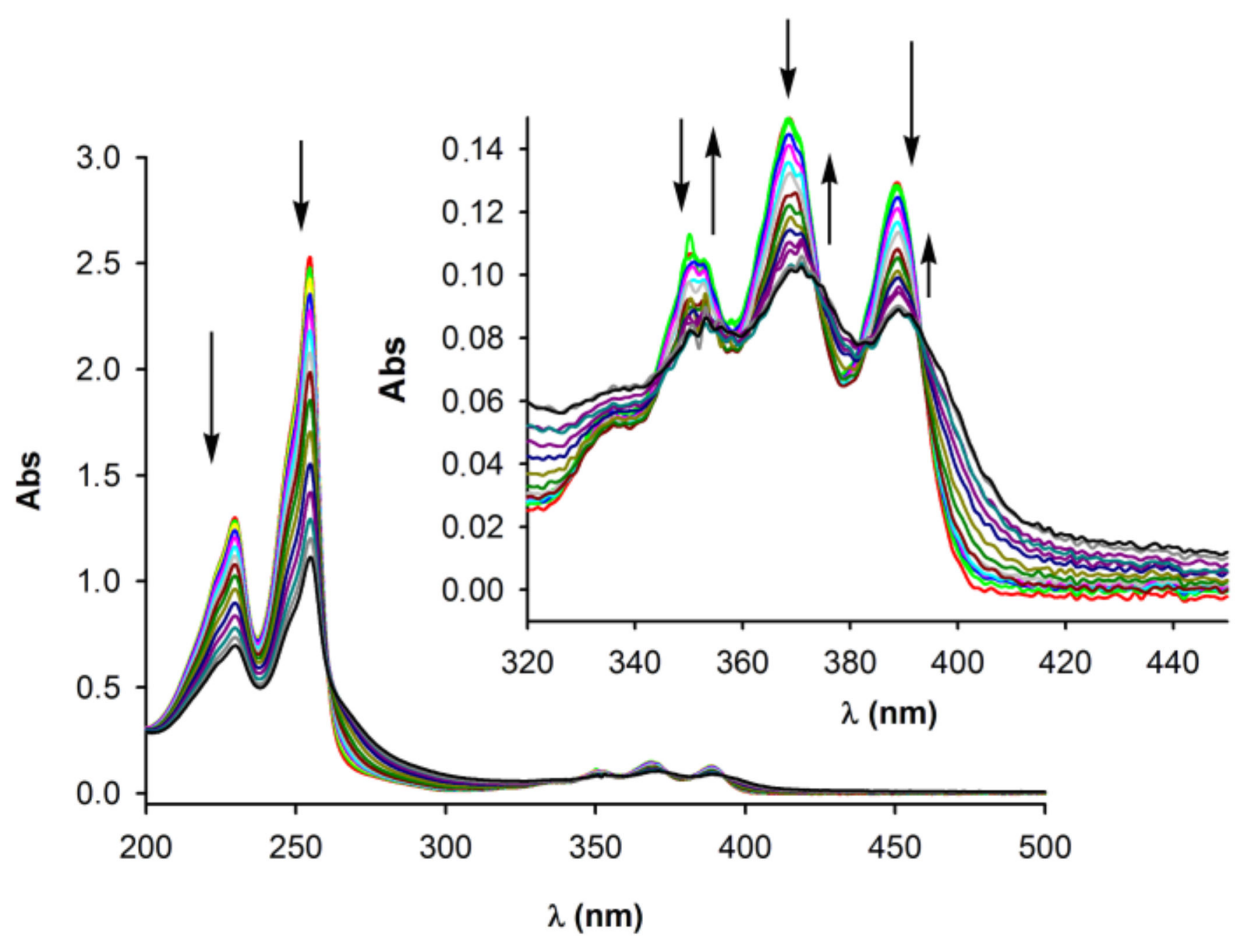
Changes in the absorption spectra of the receptor **5^2+^·2PF_6_^−^** (*c* = 1 × 10^−5^ M in CH_3_CN) upon addition of increasing amounts of H_2_PO_4_^−^ anions. Arrows indicate the absorption bands that increase or decrease during the titration.

The recognition properties of the receptor towards the above-mentioned set of anions were also evaluated by UV-vis spectroscopy. Absorption spectrum of the receptor **5^2+^·2PF_6_^−^** in CH_3_CN (*c* = 1 × 10^−5^ M) displays two intense absorption bands at λ = 230 nm (ε = 1.31 × 10^5^ M^−1^ cm^−1^) and λ = 255 nm (ε = 2.39 × 10^5^ M^−1^ cm^−1^) along with the characteristic absorption bands attributed to the anthracene moieties at λ = 334 (ε = 6 × 10^3^ M^−1^ cm^−1^), λ = 351 (ε = 11.14 × 10^4^ M^−1^ cm^−1^), λ = 370 (ε = 1.5 × 10^4^ M^−1^ cm^−1^) and λ = 390 nm (ε = 1.35 × 10^4^ M^−1^ cm^−1^). The addition of increase amounts of SO_4_^2−^, AcO^−^, HP_2_O_7_^3−^ and F^−^ anions to a solution of the receptor in CH_3_CN (*c* = 1 × 10^−5^ M) caused small perturbations in the absorption spectrum of the receptor; by contrast, the addition of H_2_PO_4_^−^ anions induced a decrease in the absorption bands at λ = 250 and λ = 255 nm while a decrease and red-shifted ∆λ = 2 nm in the anthracene absorption bands was observed. The well-defined isosbestic point observed at λ = 260, λ = 343, λ = 373, λ = 382 and λ = 390 nm indicates that a neat interconversion between the uncomplexed and complexed species occurs (Figure 4). Unfortunately, these changes do not allow an accurate determination of the association constant.

In order to obtain additional information about the binding mode of the receptor **5^2+^·2PF_6_^−^** with the previously tested anions, ^1^H-NMR titration experiments were performed. The ^1^H-NMR spectrum of the receptor in CD_3_CN/CD_3_OD (8/2) exhibit the following characteristic signals: (a) the protons of the methyl groups of the Br-imidazolium unit appears at δ = 1.86 and 2.15 ppm and the ones corresponding to the H-imidazolium moiety at δ = 2.15 and 2.59 ppm; (b) the protons of the methylenes naphthalene–CH_2_–imidazolium and imidazolium–CH_2_–anthracene appear as four different singlets around δ = 5.12 and 5.50 ppm and δ = 6.18 and 6.45 ppm, respectively; and (c) the protons of the naphthalene and the anthracene groups are embeded in the aromatic region in the range δ = 6.94–8.79 ppm.

**Figure 5:**
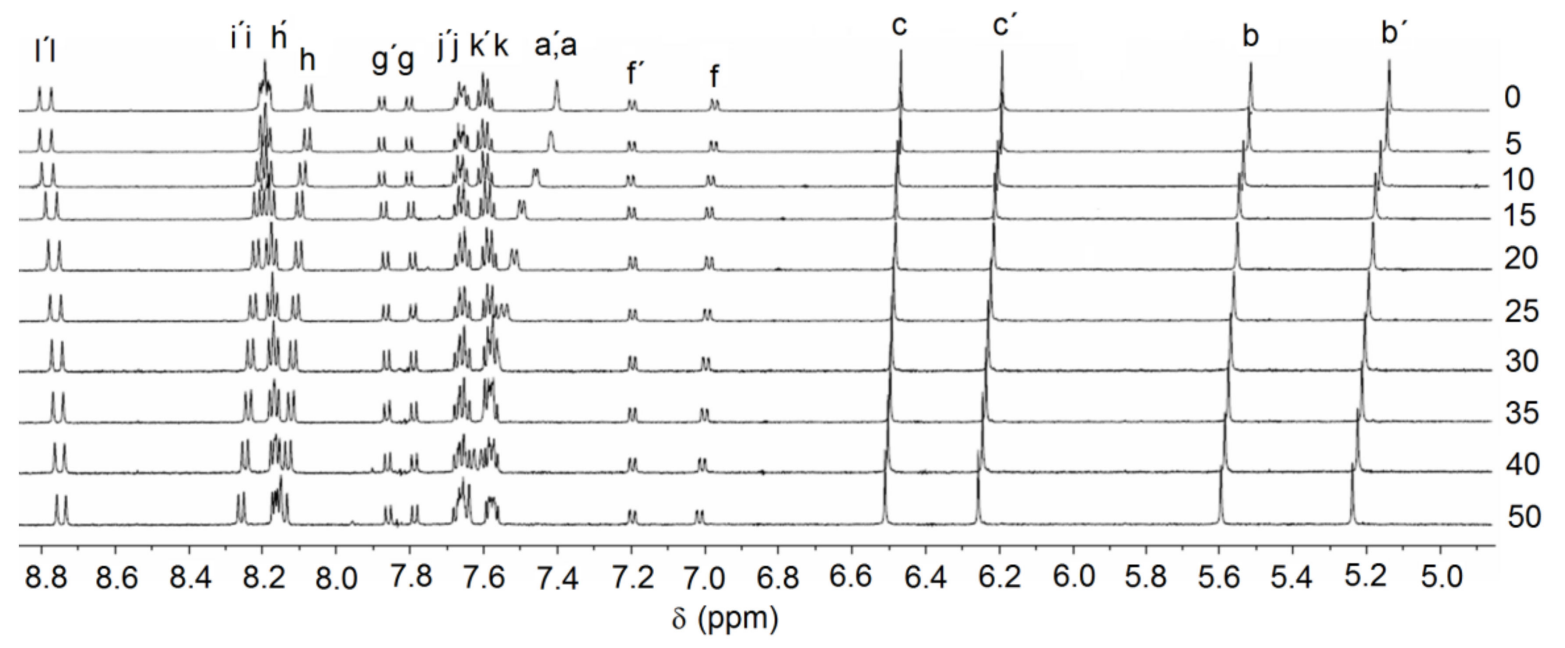
^1^H-NMR spectral changes observed in the receptor **5^2+^·2PF_6_^−^** in CD3CN/CD3OD (8/2, *v*/*v*) during the addition from 0 (top) to 50 equiv. (bottom) of H_2_PO_4_^−^ anions.

The addition of increase amounts of H_2_PO_4_^−^ (Figure 5) anions to a solution of the receptor **5^2+^·2PF_6_^−^** in CD_3_CN/CD_3_OD (8/2) induced the splitting and a significant downfield shifts of the inner naphthalene protons *H_a,a’_* (∆δ ~ 0.23 ppm). The methylene protons *H_b_*, *H_b’_*, *H_c_* and *H_c’_* were also downfield shifted; ∆δ = 0.09, 0.11, 0.05 and 0.07 ppm, respectively. Unfortunately the observation of the downfield shift of the H-imidazolium proton was not detected due to the signal of this proton within the aromatic region; nevertheless, the simultaneous participation in the anion binding process of the H and the Br sited at C-2 of the H-imidazolium and Br-imidazolium rings, respectively, was supported by the upfield shift observed in the methyl protons of the Br-imidazolium ring *H_d_* (∆δ = −0.16 ppm) and in the methyl protons *H_e’_* of the H-imidazolium ring (∆δ = −0.10 ppm) (Figure S13).

Similar changes were observed with the presence of SO_4_^2−^ anions but to a lesser extent than those produced with H_2_PO_4_^−^ anions (Figure S10).

**Scheme 2:**
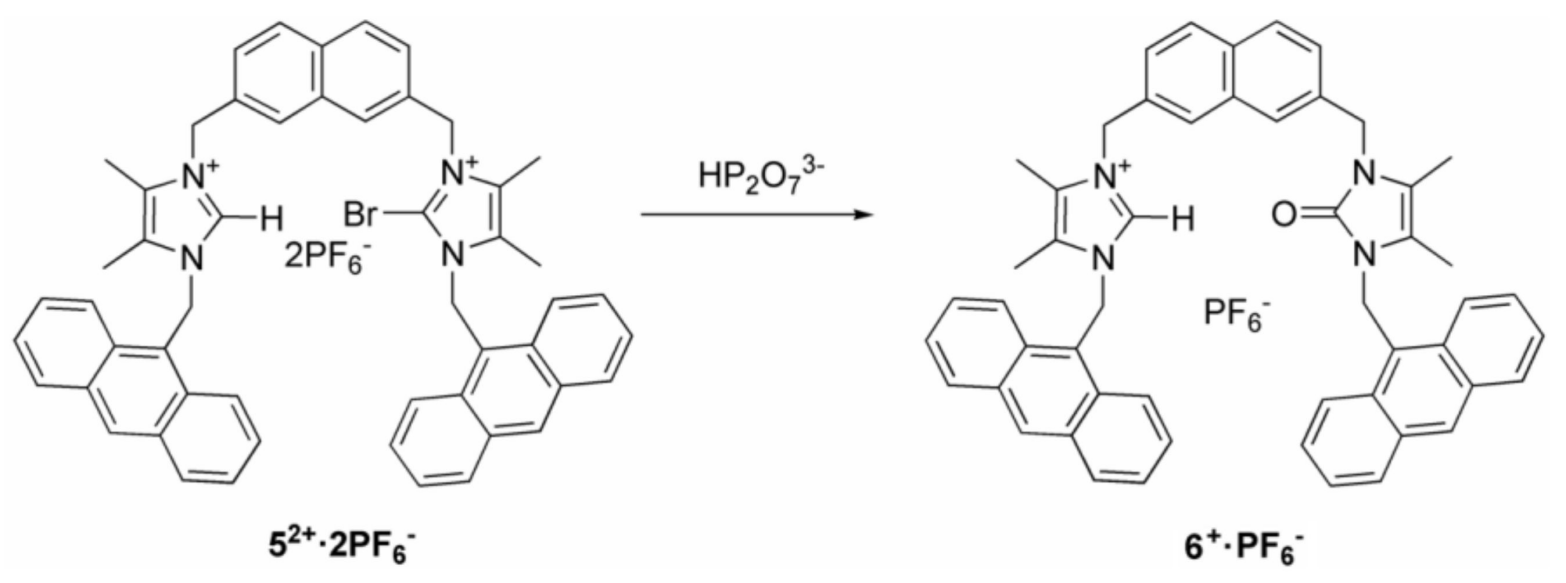
Synthesis of imidazolone **6^2+^·PF_6_^−^** from the receptor **5^2+^·2PF_6_^−^** by the HP_2_O_7_^3−^ anion.

The addition of increasing amounts of HP_2_O_7_^3−^ (Figure S11) and F^−^ anions (Figure S12) to a solution of the hydrogen and halogen bonding receptor **5^2+^·2PF_6_^−^** produce different changes in the ^1^H-NMR than those previously described for H_2_PO_4_^−^ and SO_4_^2−^ anions. In these cases, the addition of HP_2_O_7_^3−^ and F^−^ anions up to 1 equiv. promote the downfield shift of the internal naphthalene protons *H_a,a’_* (∆δ ~ 0.09 ppm) as well as the methylene protons *H_b_*, *H_b’_*, *H_c_* and *H_c’_* (∆δ ~ 0.03 ppm), suggesting that a recognition processes is taking place. However, the addition of more than 1 equiv. of HP_2_O_7_^3-^ induced the appearance of new signals and the progressive disappearance of the signals assigned to the complexes formed between the receptor **5^2+^·2PF_6_^−^** with HP_2_O_7_^3−^ or F^−^ anions. After addition of 2 equiv. of anion, the ^1^H-NMR spectrum showed only one set of signals. The titration was also followed by mass spectrometry. The results indicate that a debromination process is taking place in the bromo-imidazolium ring by the formation of an imidazolone ring to generate the new compound **6^2+^·PF_6_^−^** (Scheme 2). Interestingly the H-imidazolium ring remains unperturbed in the presence of HP_2_O_7_^3−^ and F^−^ anions. These results are consistent with those reported previously for the halogen and hydrogen bonding receptor **7^2+^·2PF_6_^−^** and **8^2+^·2PF_6_^−^**, respectively [10] and dramatically reveal the lability of the C2–X bond of the haloimidazolium ring that undergoes cleavage in the presence of basic anions, which constitutes a clear and important limitation for the use of such kind of receptors against basic anions.

The association constants calculated from the ^1^H-NMR titration data using the Dynafit program [15] are shown in Table 2.

A comparative study of the association constants obtained for the receptor **5^2+^·PF_6_^−^** with those reported for the halogen bonding receptor **7^2+^·2PF_6_^−^** and the hydrogen bonding receptor **8^2+^·2PF_6_^−^** in the competitive mixture CD_3_CN/CD_3_OD 8:2 *v*/*v* indicates that the receptors bind the SO_4_^2−^ anions following the trend **7^2+^·2PF_6_^−^** (XB receptor) > **5^2+^·PF_6_^−^** (XB and HB) > **8^2+^·2PF_6_^−^** (HB). Thus, the presence of halogen bonding interactions increases the strength of the receptors for SO_4_^2−^ anion. Interestingly, the association constant between the receptor **5^2+^·PF_6_^−^** and SO_4_^2−^ anions is 18 times higher than with the H_2_PO_4_^−^ anion, while higher association constants for the receptors **7^2+^·2PF_6_^−^** and **8^2+^·2PF_6_^−^** were found for H_2_PO_4_^−^ anion, and therefore the halogen and hydrogen bonding receptor **5^2+^·PF_6_^−^** is the receptor which shows the highest selectivity for SO_4_^2−^ anions.

## 3. Experimental Section

### 3.1. General Comments

All reactions were carried out using solvents that were dried by routine procedures. All melting points were determined by means of a Kofler hot-plate melting-point apparatus (Wagner & Munz, München, Germany) and are uncorrected. Solution ^1^H- and ^13^C-spectra were recorded with Bruker 200, 300, 400, or 600 MHz spectrometers (Bruker Corporation, Billerica, MA, USA). The following abbreviations have been used to state the multiplicity of the signals: s (singlet), m (multiplet), and q (quaternary carbon atom). Chemical shifts (δ) in the ^1^H- and ^13^C-NMR spectra are referenced to tetramethylsilane (TMS). UV−vis and fluorescence spectra were carried out in the solvents and concentrations stated in the text and in the corresponding figure captions, using a dissolution cell with 10 mm path length, and they were recorded with the spectra background corrected before and after sequential additions of different aliquots of anions. Quantum yield values were measured with respect to anthracene as the standard (Φ = 0.27 ± 0.01) using the equation:

Φ_x_/Φ_s_ = (S_x_/S_s_)((1 − 10^−As^)/(1 − 10^−Ax^))(n_s_^2^/n_x_^2^) (1)

where x and s indicate the unknown and standard solution, respectively, Φ is the quantum yield, S is the area under the emission curve, A is the absorbance at the excitation wavelength and n is the refractive index. Mass spectra were recorded with a MLC-MS TOF 6220 (Agilent Technologies Germany, Waldbronn, Germany).

### 3.2. General Procedure for Synthesis of Compounds ***2*** and ***4***

To a solution of 4,5-dimethyl-1*H*-imidazol (0.27 g, 2.81 mmol) or 2-bromo-4,5-dimethyl-1*H*-imidazol (0.3 g, 1.71 mmol) in CH_3_CN (120 mL) was added dropwise an aqueous solution of 1 M NaOH (3.27 mmol). The reaction medium was stirred for 10 min and after this time, 9-(bromomethyl)anthracene (0.5 g, 1.75 mmol) was added in one portion; the resulting mixture was stirred at 0 °C for 30 min in the case of compound **2** and 2h at room temperature for compound **4**. The resulting precipitate was separated by filtration and purified by silica gel column chromatography (CH_2_Cl_2_/CH_3_OH 95:5) to yield compound **2**. In the case of compound **4**, the resulting precipitate was filtered and dissolved in CH_2_Cl_2_ (50 mL) and then washed with water (2 × 50 mL). The organic solvent was collected and dried with anhydrous Na_2_SO_4_. After filtration, the solvent was removed under reduced pressure to yield the desired compound.

*1-((anthracen-9-yl)methyl)-4,5-dimethyl-1H-imidazole* (**2**).Yellow solid, yield 19%, ^1^H-NMR (CDCl_3_, 400 MHz) δ = 8.55 (s, 1H); 8.06–8.01 (m, 4H), 7.53–7.46 (m, 4H); 6.56 (s, 1H); 5.76 (s, 2H); 2.41 (s, 3H); 2.18 (s, 3H); ^13^C-NMR (CDCl_3_, 100 MHz) δ = 134.3; 133.6; 131.4; 130.9; 129.3; 127.3; 125.3; 124.1; 123.1; 122.2; 41.7; 12.7; 9.0; MS (ESI, *m*/*z*): 287.10 [M + H]^+^.

*1-((anthracen-9-yl)methyl)-2-bromo-4,5-dimethyl-1H-imidazole* (**4**). Yield 48%, ^1^H-NMR (CDCl_3_, 400 MHz) δ = 8.48 (s, 1H); 8.10–8.05 (m, 4H), 7.57–7.48 (m, 4H); 6.06 (s, 2H); 1.97 (s, 3H); 1.22 (s, 3H); ^13^C-NMR (CDCl_3_, 100 MHz) δ = 135.6; 132.1; 131.6; 130.4; 130.3; 128.1; 127.5; 126.1; 125.1; 124.1; 117.1; 45.4; 12.9; 10.9; MS (ESI, *m*/*z*): 364.08 [M + H]^+^.

### 3.3. Synthesis of Compound ***3^+^·Br^−^***

To a solution of compound **2** (0.25 g, 0.87 mmol) in CH_3_CN (180 mL), 2,7-bis(bromomethyl)naphthalene (0.65 g, 2 mmol) was added in one portion. The mixture was stirred for 90 min at 60 °C; the volatile compounds were removed under vacuum and the resulting residue was purified by silica gel column chromatography (CH_2_Cl_2_/CH_3_OH 9:1) to yield compound **3** as bromide salt. Yield 63%, ^1^H-NMR (CDCl_3_, 400 MHz) δ = 9.54 (s, 1H); 8.57 (s, 1H), 8.35 (d, 2H, *J* = 9 Hz), 8.06 (d, 2 H, *J* = 9 Hz), 7.73–7.66 (m, 5H); 7.40-7.46 (m, 4H), 6.45 (s, 2H); 5.48 (s, 2H); 4.71 (s, 1H), 4.61 (s, 1H), 2.03 (s, 3H); 1.66 (s, 3H); ^13^C-NMR (CDCl_3_, 75 MHz) δ = 136.0; 135.4; 132.8; 132.5; 131.2; 131.0; 130.7; 130.6; 129.6; 128.9; 128.4; 128.2; 127.9; 127.6; 127.4; 126.4; 125.5; 125.0; 122.9; 121.3; 50.9; 45.1; 33.6; 9.7; 8.7; MS (ESI, *m*/*z*): 519.20 [M]^+^.

### 3.4. Synthesis of Receptor ***5^2+^·2Br^−^***

To a solution of compound **3^+^·Br^−^** (0.3 g, 0.5 mmol) in acetonitrile (200 mL), compound **4** (0.36 g, 0.98 mmol) was added. The reaction mixture was stirred at 60 °C for 48 h. The solvent was then removed under reduced pressure and the crude was purified by silica gel column chromatography (CH_2_Cl_2_/CH_3_OH 9:1) to yield the desired compound as bromide salt. Yield 25%, ^1^H-NMR (*d*_6_-DMSO, 300 MHz) δ = 8.90 (s, 1H); 8.83 (s, 1H), 8.38 (d, 2H, *J* = 9 Hz), 8.30 (s, 1H), 8.26–8.17 (m, 6H), 7.95 (d, 1H, *J* = 9 Hz), 7.88 ( d, 1H, *J* = 8 Hz), 7.70–7.55 (m, 10H), 7.23 (dd, 1H, *J* = 9 Hz, *J* = 0.5 Hz), 7.00 (dd, 1H, *J* = 9 Hz, *J* = 0.5 Hz), 6.62 (s, 2H), 6.37 (s, 2H), 5.66 (s, 2H), 5.34 (s, 2H); 2.59 (s, 3H), 2.17 (s, 3H); 2.13 (s, 3H), 1.82 (s, 3H); ^13^C-NMR (*d*_6_-DMSO, 75 MHz) δ = 135.6; 134.4; 133.8; 133.3; 132.6; 132.3; 132.1; 131.8; 131.5; 131.3; 131.1; 130.9; 130.2; 130.1; 129.1; 129.0; 128.3; 127.2; 127.1; 126.9; 126.4; 126.2; 125.8; 125.0; 124.7; 124.3; 123.4; 122.9; 51.6; 50.8; 48.5; 45.1; 11.4; 10.4; 9.5; MS (ESI, *m*/*z*): 885.2 [M^2+^ + Br^−^]^+^.

### 3.5. Synthesis of Receptor ***5^2+^·2PF_6_^−^***

A solution of the bis-imidazolium receptor as the bromide salt **5^2+^·2Br^−^** (0.1 g, 0.1 mmol) in CH_2_Cl_2_ (20 mL) was washed with a saturated solution of NH_4_PF_6_ (4 × 20 mL) for 30 min. The organic layer was separated, dried over anhydrous Na_2_SO_4_, filtered and evaporated under reduced pressure, yielding the desired receptor. Yield 62%, ^1^H-NMR (*d*_6_-DMSO, 400 MHz) δ = 8.90 (s, 1H); 8.83 (s, 1H), 8.33 (d, 2H, *J* = 8 Hz), 8.30 (s, 1H), 8.26 (d, 2H, *J* = 8 Hz), 8.22 (d, 2H, *J* = 8 Hz), 8.14 (d, 2H, *J* = 8 Hz), 7.94 (d, 1H, *J* = 8 Hz), 7.87 (d, 1H, *J* = 8 Hz), 7.70-7.58 (m, 8H), 7.53 (s, 1H), 7.49 (s, 1H), 7.19 (dd, 1H, *J* = 8 Hz, *J* = 0.5 Hz), 6.95 (dd, 1H, *J* = 8 Hz, *J* = 0.5 Hz), 6.57 (s, 2H), 6.33 (s, 2H), 5.65 (s, 2H), 5.32 (s, 2H); 2.58 (s, 3H), 2.17 (s, 3H); 2.13 (s, 3H), 1.40 (s, 3H); ^13^C-NMR (*d*_6_-DMSO, 100 MHz) δ = 134.0; 132.9; 132.3; 132.0; 131.9; 131.2; 130.9; 130.8; 130.6; 130.4; 130.1; 129.7; 129.5; 128.9; 128.7; 128.6; 126.9; 125.9; 125.6; 125.5; 125.0; 124.8; 124.4; 123.4; 123.1; 122.8; 121.8; 121.3; 50.2; 48.4; 46.8; 43.5; 9.8; 8.9; 8.4; 7.9; MS (ESI, *m*/*z*): 951.2 [M^2+^ + PF**_6_^−^**]^+^.

## 4. Conclusions

We have reported the synthesis of a novel anion sensor **5^2+^·2PF_6_^−^** which binds anions by the cooperative action of halogen- and hydrogen-bonding interactions. Several anion-binding experiments have been carried out in order to make a comparative study of the sensing capabilities of the novel XB and HB receptor **5^2+^·2PF_6_^−^** regarding the XB and HB analogues **7^2+^·2PF_6_^−^** and **8^2+^·2PF_6_^−^**, respectively. Evaluation of the sensing properties by fluorescence in CH_3_CN reveals important similarities in the sensing behaviour between the halogenated receptor **7^2+^·2PF_6_^−^** and the mixed XB and HB receptor **5^2+^·2PF_6_^−^**. The XB and HB receptor **5^2+^·2PF_6_^−^** acts as a selective fluorescent molecular sensor for H_2_PO_4_^−^ anion, because it is the unique anion which promotes the appearance of the anthracene excimer emission band. In addition, the presence of the HP_2_O_7_^3−^ anion produces the selective transformation in the corresponding mono-imidazolone **6^2+^·2PF_6_^−^** after debromination of the Br-imidazolium ring while the H-imidazolium ring remained unperturbed. The analysis of the obtained association constant values obtained from the ^1^H-NMR titration experiments in CD_3_CN/MeOD (8/2) reveals that the selectivity showed for the mixed XB and HB receptor **5^2+^·2PF_6_^−^** for SO_4_^2−^ anions is higher than the ones observed for the halogen or hydrogen bonding analogues. The magnitude of the association constant with SO_4_^2−^ anions of the mixed XB and HB receptor **5^2+^·2PF_6_^−^** is intermediate between the halogen bonding receptor **7^2+^·2PF_6_^−^** and the hydrogen bonding receptor **8^2+^·2PF_6_^−^** in this competitive methanolic medium. These results highlight the importance of the combinations of different non-covalent interactions in selective anion recognition and sensing.

## Figures and Tables

**Table 1:** Association constants for receptor **5^2+^·PF_6_^−^**, **7^2+^·2PF_6_^−^** and **8^2+^·2PF_6_^−^** with H_2_PO_4_^−^, SO_4_^2−^ and AcO^−^ anions in CH_3_CN measured by fluorescence technique. Errors (in percent) are given in parentheses.

Receptor	H_2_PO_4_^−^	SO_4_^2−^	AcO^−^
**5^2+^·PF_6_^−^**	*β* = 5.9 × 10^10^ M^−2^ (15)	*K* = 5.7 × 10^4^ M^−1^ (14)	*β* = 2.16 × 10^9^ M^−2^ (10)
**7^2+^·2PF_6_^−^**	*β* = 5.0 × 10^11^ M^−2^ (16)	*K* = 8.0 × 10^4^ M^−1^ (14)	-
**8^2+^·2PF_6_^−^**	*β* = 9.8 × 10^11^ M^−2^ (13)	*K* = 1.4 × 10^6^ M^−1^ (6)	-

**Table 2:** Association constants for receptors **5^2+^·2PF_6_^−^**, **7^2+^·2PF_6_^−^** and **8^2+^·2PF_6_^−^** with H_2_PO_4_^−^ and SO_4_^2−^ anions in CD_3_CN/CD_3_OD 8:2 *v*/*v* measured using the ^1^H-NMR technique. Errors (in percent) are given in parentheses.

Receptor	H_2_PO_4_^−^	SO_4_^2−^	*K*(SO_4_^2−^)/*β*(H_2_PO_4_^−^)
**5^2+^·PF_6_^−^**	*β* = 1.37 × 10^2^ M^−2^ (4)	*K* = 2.6 × 10^3^ M^−1^ (4)	18
**7^2+^·2PF_6_^−^**	*β* = 9.9 × 10^3^ M^−2^ (3)	*K* = 5.5 × 10^3^ M^−1^ (4)	0.55
**8^2+^·2PF_6_^−^**	*β* = 5.6 × 10^3^ M^−2^ (4)	*K* = 2.2 × 10^3^ M^−1^ (4)	0.39

## Supplementary Materials

The supplementary materials are available online. ^1^H- and ^13^C-NMR spectra of the compound, Figures S1–S3: Changes in the emission spectrum of **5^2+^·2PF_6_^−^** upon addition of F**^−^** and AcO**^−^** anions, Figures S4–S6: Calculation of the detection limits, Figures S7–S9: Job´s Plot experiments, Figures S10–S13: ^1^H-NMR spectral changes of the receptor **5^2+^·2PF_6_^−^** during the addition of SO_4_^2−^, HP_2_O_7_^3−^ and F^−^ anions.
