# Supplementary material for: Modulation of the Selectivity in Anions Recognition Processes by Combining Hydrogen- and Halogen-Bonding Interactions
Source: Molecules. 2017 Dec 20;22(12):2273. doi: 10.3390/molecules22122273 (PMC6149874; doi:10.3390/molecules22122273)
Supplement: Supplementary file 1 [file molecules-22-02273-s001.pdf]

Supporting Information (SI)

Modulation of the selectivity in anions recognition processes by complexing hydrogen- and halogen- bonding interactions

Fabiola Zapata, Sergio J. Benítez-Benítez, Paula Sabater, Antonio Caballero,* and Pedro Molina *

Departamento de Química Orgánica. Facultad de Química. Universidad de Murcia. Campus de Espinardo, E-30100 Murcia, Spain.

E-mail: pmolina@um.es; antocaba@um.es

Table of contents

^{1}H- and ^{13}C-NMR spectra of compounds **2-5** S3

Figure S1. Changes in the fluorescence emission of $\mathbf{5^{2+}\cdot 2PF_6^-}$ in CH_3CN upon addition of F^- anions from 0 to 1.8 equiv S8

Figure S2. Changes in the fluorescence emission of $\mathbf{5^{2+}\cdot 2PF_6^-}$ in CH_3CN upon addition of F^- anions from 1.8 to 5 equiv S8

Figure S3. Changes in the fluorescence emission of $\mathbf{5^{2+}\cdot 2PF_6^-}$ in CH_3CN upon addition of AcO^- anion. S9

Figure S4. Semilogarithmic plot for determining the detection limit of $\mathbf{5^{2+}\cdot 2PF_6^-}$ towards $H_2PO_4^-$. S9

Figure S5. Semilogarithmic plot for determining the detection limit of
5^{2+}·$2PF_6^-$ towards AcO^-. S10
Figure S6. Semilogarithmic plot for determining the detection limit of
5^{2+}·$2PF_6^-$ towards SO_4^{2-}. S10
Figure S7. Job´s plot for **5^{2+}·$2PF_6^-$** and $H_2PO_4^{2-}$ S11
Figure S8. Job´s plot for **5^{2+}·$2PF_6^-$** and SO_4^{2-} S11
Figure S9. Job´s plot for **5^{2+}·$2PF_6^-$** and AcO^- S12
Figure S10. ^{1}H NMR spectral changes of the receptor **5^{2+}·$2PF_6^-$** during the addition of SO_4^{2-} anion. S12
Figure S11. ^{1}H NMR spectral changes of the receptor **5^{2+}·$2PF_6^-$** during the addition of $HP_2O_7^{3-}$ anion. S13
Figure S12. ^{1}H NMR spectral changes of the receptor **5^{2+}·$2PF_6^-$** during the addition of F^- anion. S14
Figure S13. ^{1}H NMR spectral changes observed in the methyl protons of the receptor **5^{2+}·$2PF_6^-$** during the addition of $H_2PO_4^-$ ions. S14
Figure S14. Titration profile of the receptor **5^{2+}·$2PF_6^-$** and $H_2PO_4^-$ from the ^{1}H-NMR experiments S15
Figure S15. Titration profile of the receptor **5^{2+}·$2PF_6^-$** and SO_4^{2-} from the ^{1}H-NMR experiments S15
Figure S16. Mass spectrum of the imidazolone **6^+·PF_6^-** S16

Compound 2

^{1}H-NMR ($CDCl_3$, 400 MHz)

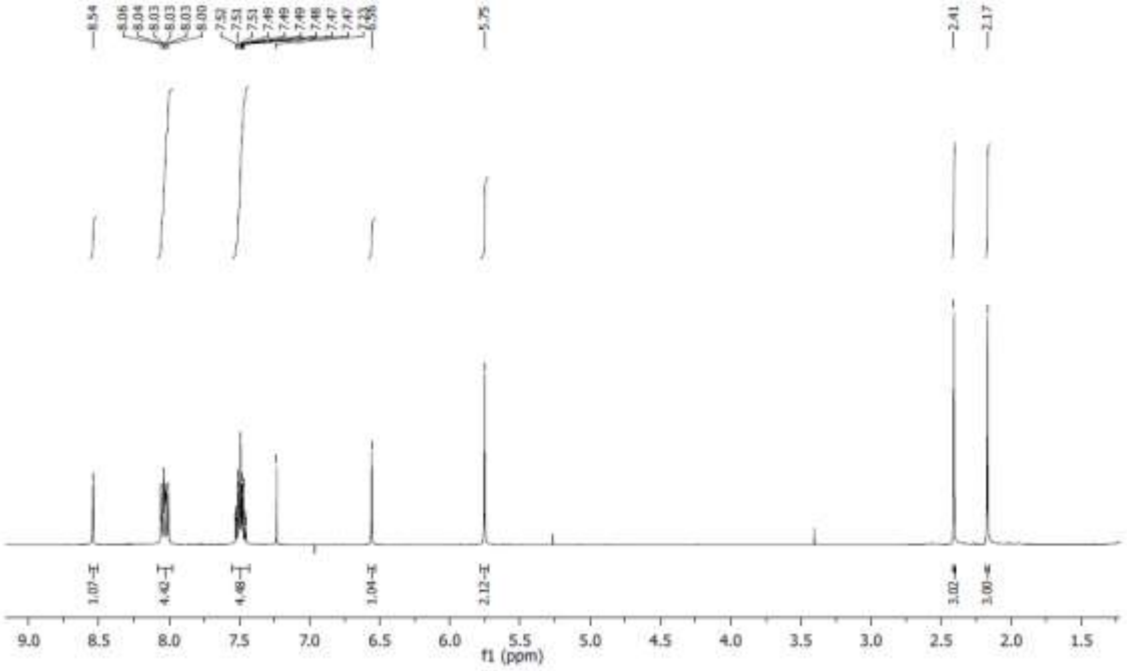

^{13}C NMR (100 MHz, $CDCl_3$)

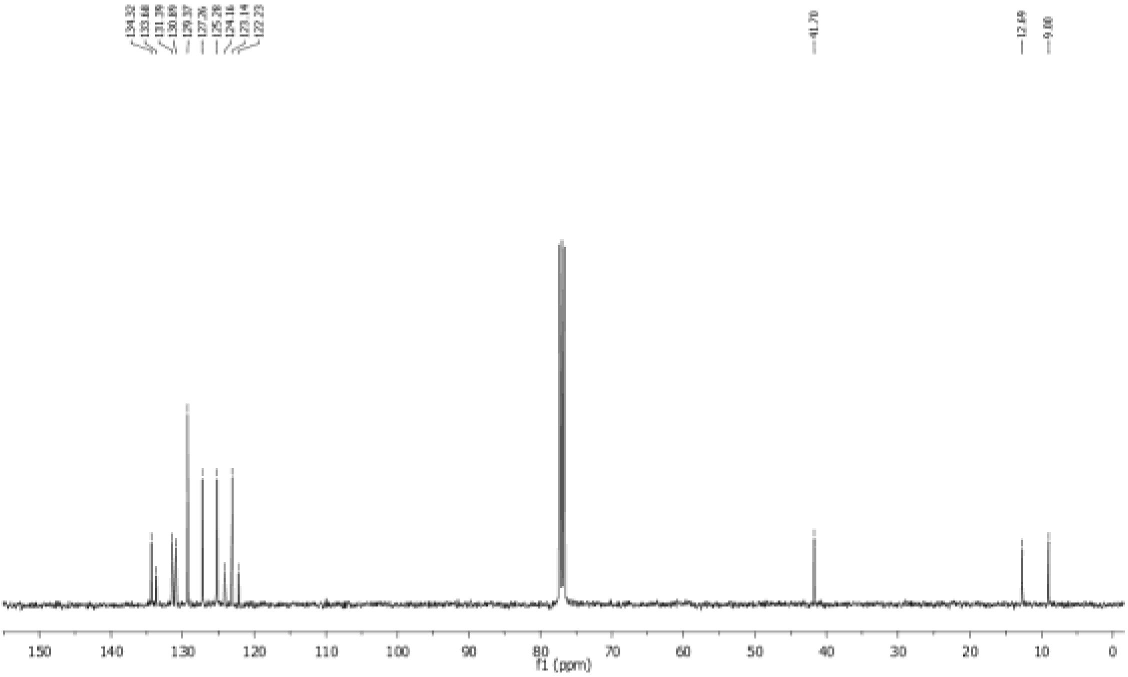

Compound 3⁺·Br⁻

^{1}H-NMR (CDCl$_3$, 400 MHz)

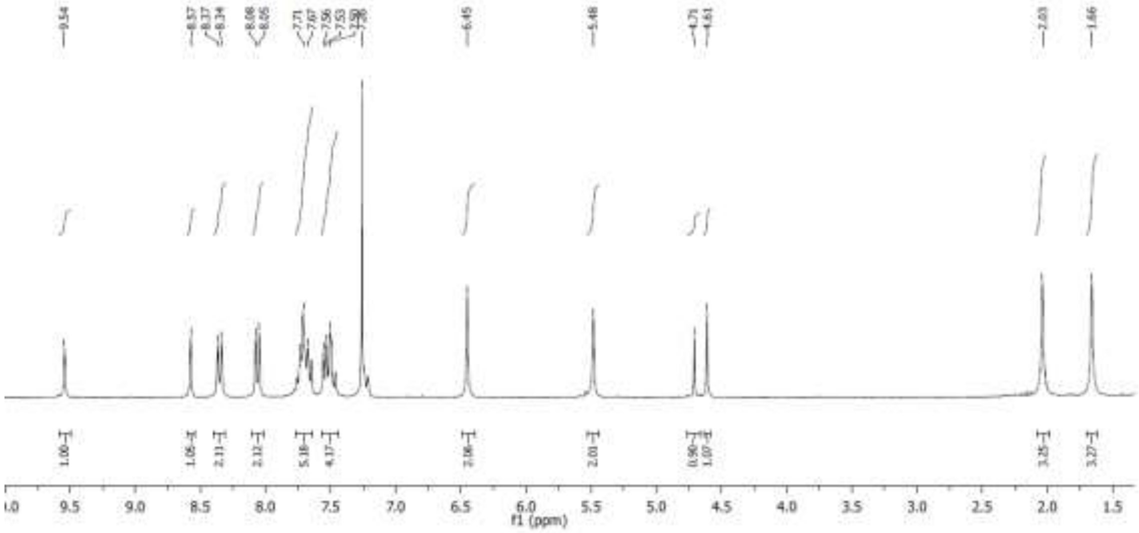

^{13}C-NMR (CDCl$_3$, 75 MHz)

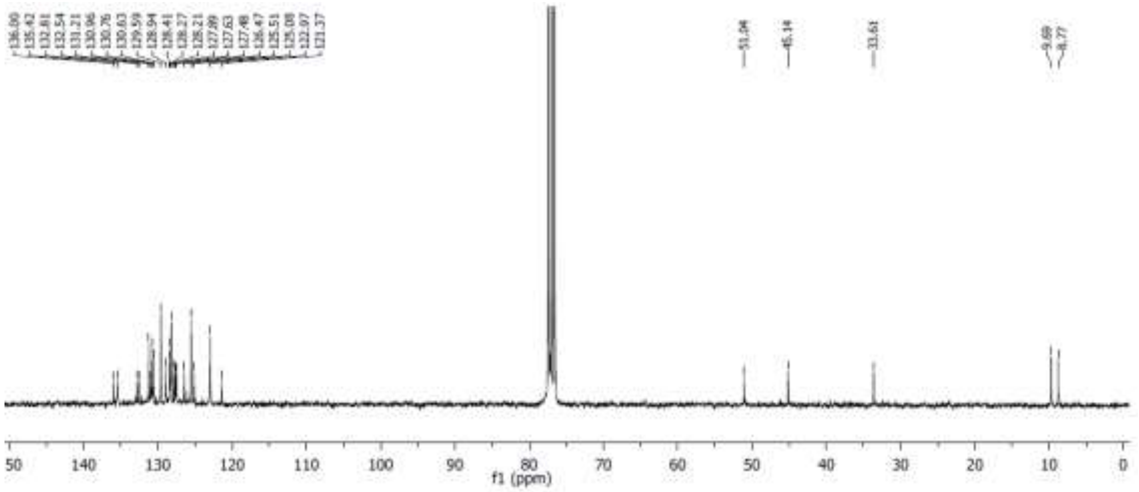

Compound 4

^{1}H-NMR ($CDCl_3$, 400 MHz)

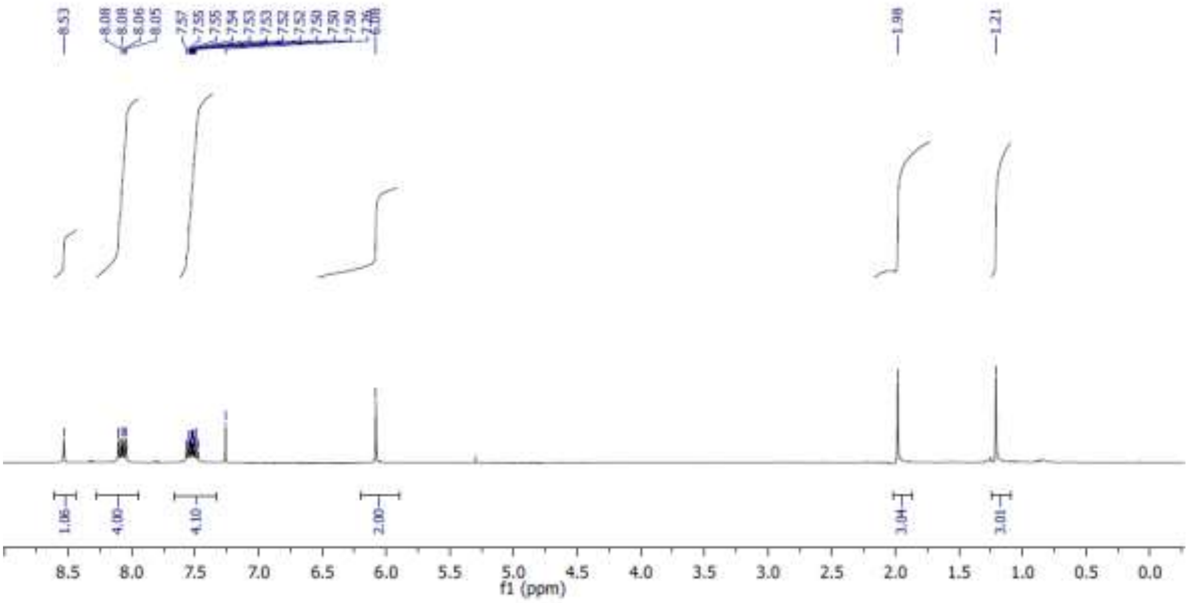

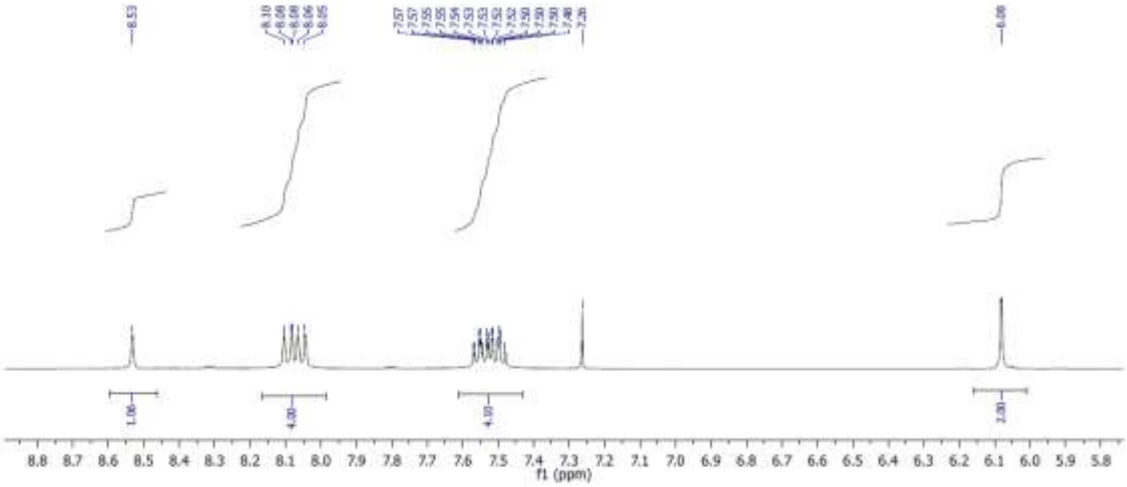

^{13}C-NMR ($CDCl_3$, 100 MHz)

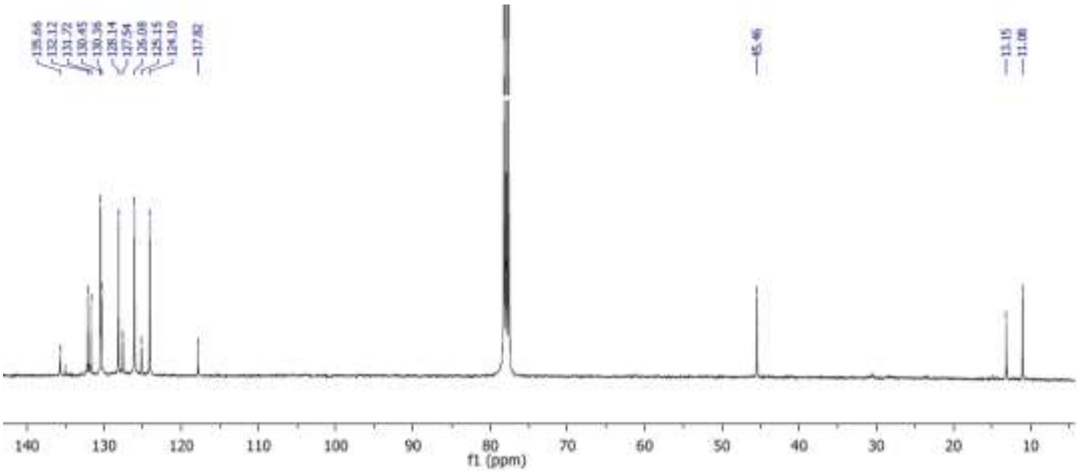

Compound $5^{2+}\cdot 2Br^{-}$

^{1}H-NMR (DMSO, 400 MHz)

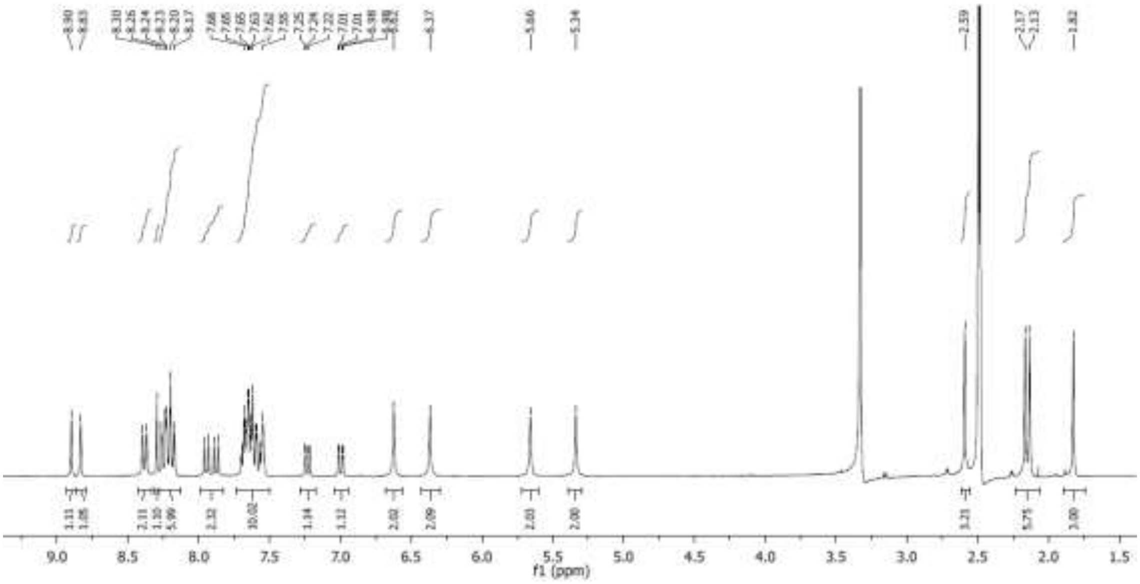

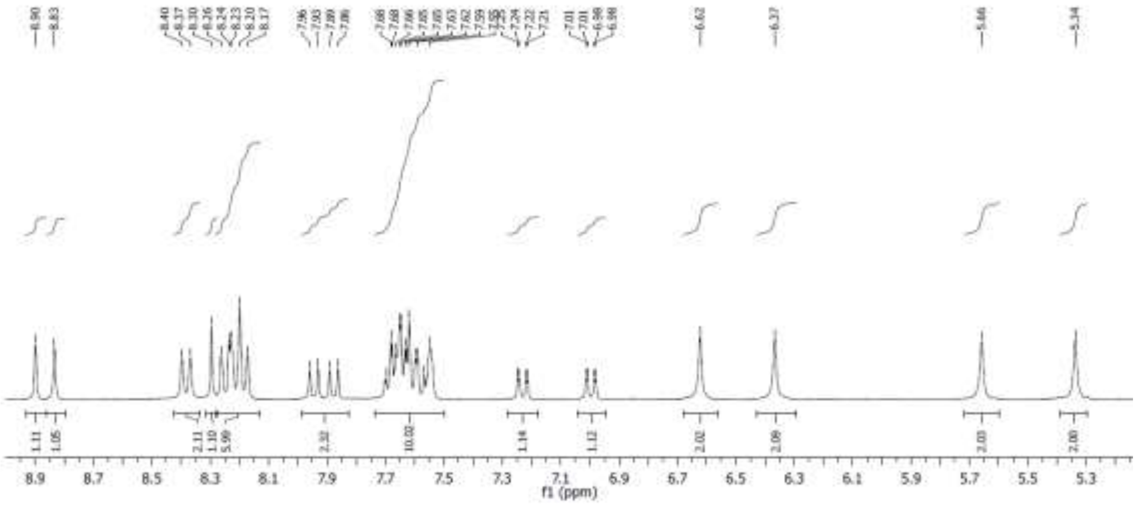

^{13}C-NMR (DMSO, 75 MHz)

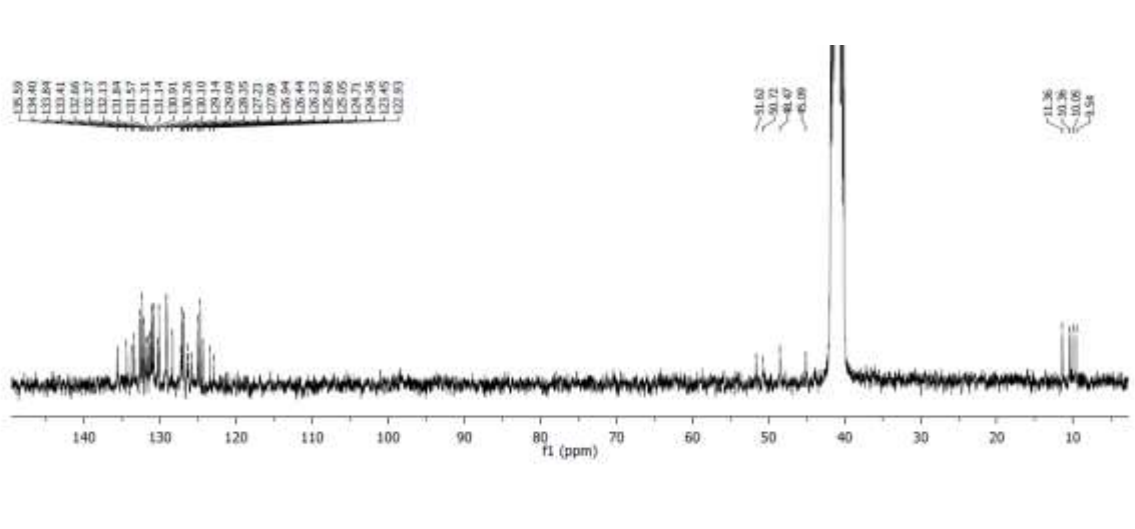

Compound $5^{2+}\cdot 2PF_6^-$

^{1}H-NMR (DMSO, 400 MHz)

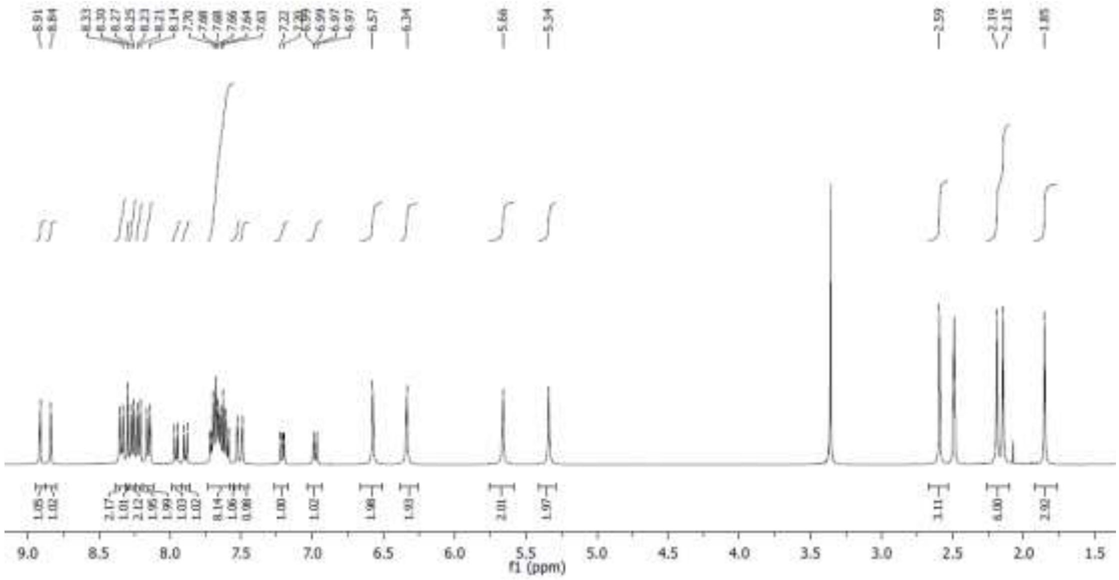

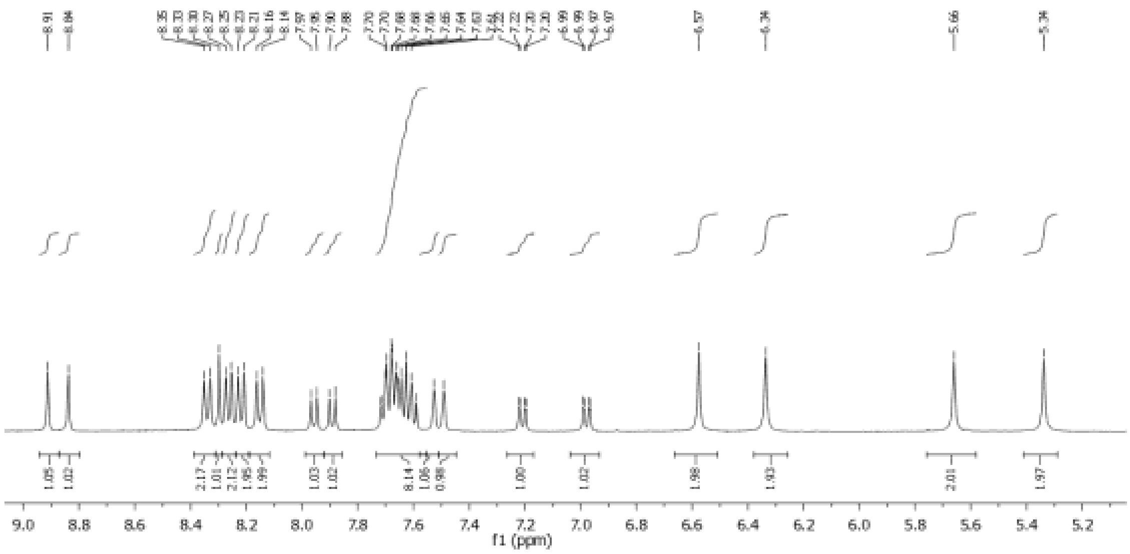

^{13}C-NMR (DMSO, 100 MHz)

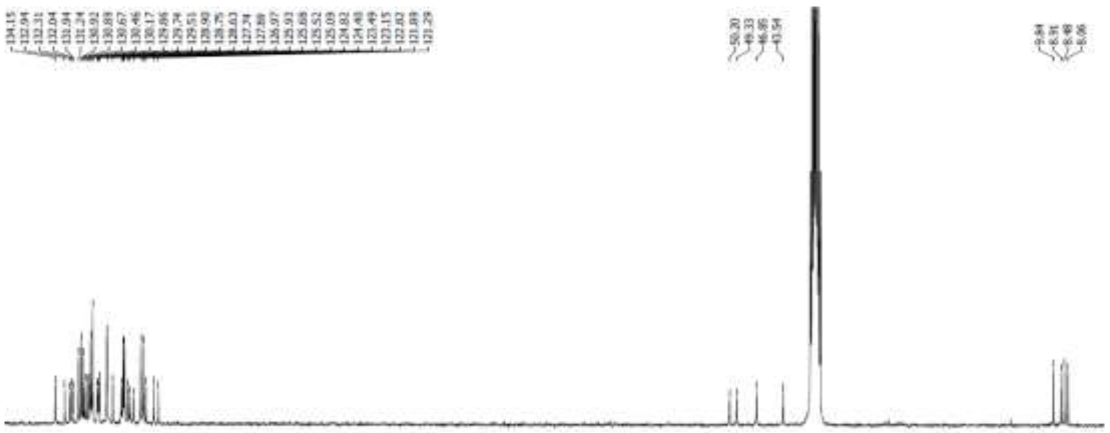

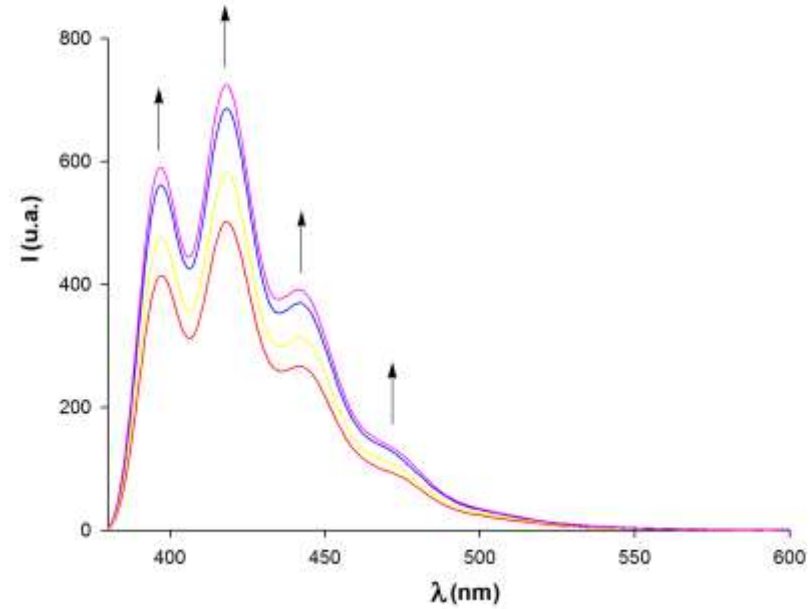

Figure S1. Changes in the fluorescence spectra of receptor $\mathbf{5^{2+}\cdot 2PF_6^-}$ (c = $1\cdot 10^{-5}$ M in CH_3CN) upon addition of F^- anions from 0 to 1.8 equiv at 20 °C.

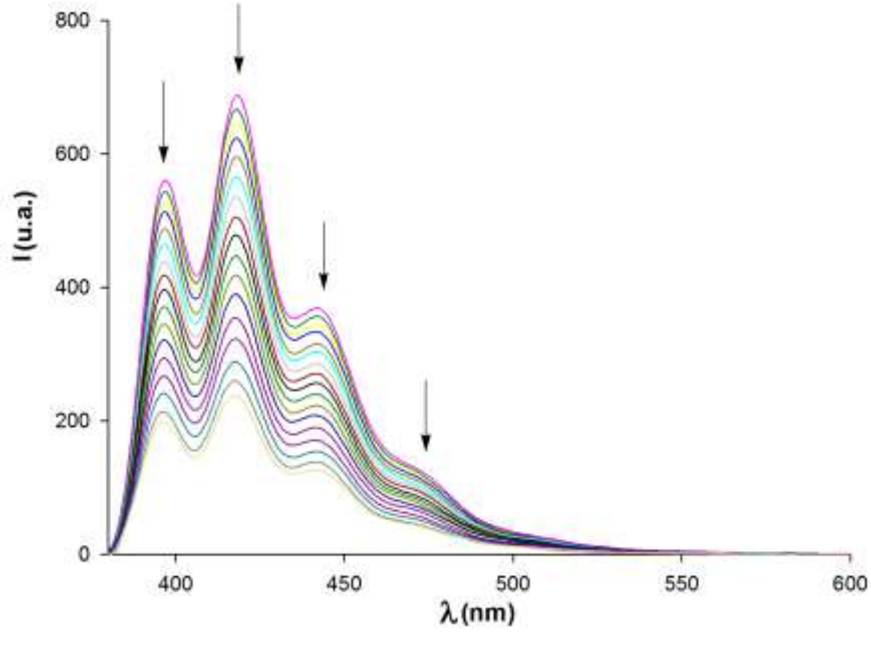

Figure S2. Changes in the fluorescence spectra of receptor $\mathbf{5^{2+}\cdot 2PF_6^-}$ (c = $1\cdot 10^{-6}$ M in CH_3CN) upon addition of F^- anions from 1.8 to 5 equiv at 20 °C.

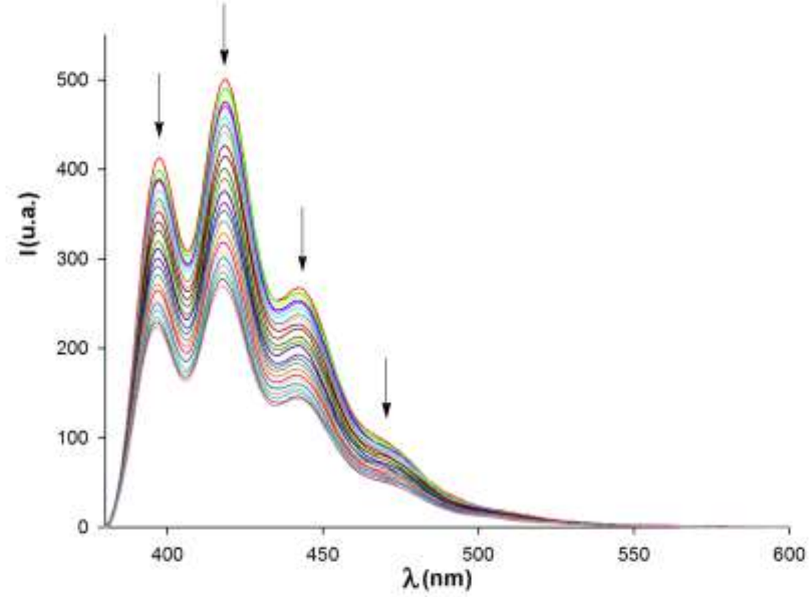

Figure S3. Changes in the fluorescence spectra of receptor $\mathbf{5^{2+}{\cdot}2PF_6^-}$ (c = $1{\cdot}10^{-6}$ M in CH_3CN) upon addition of AcO^- anions at 20 °C.

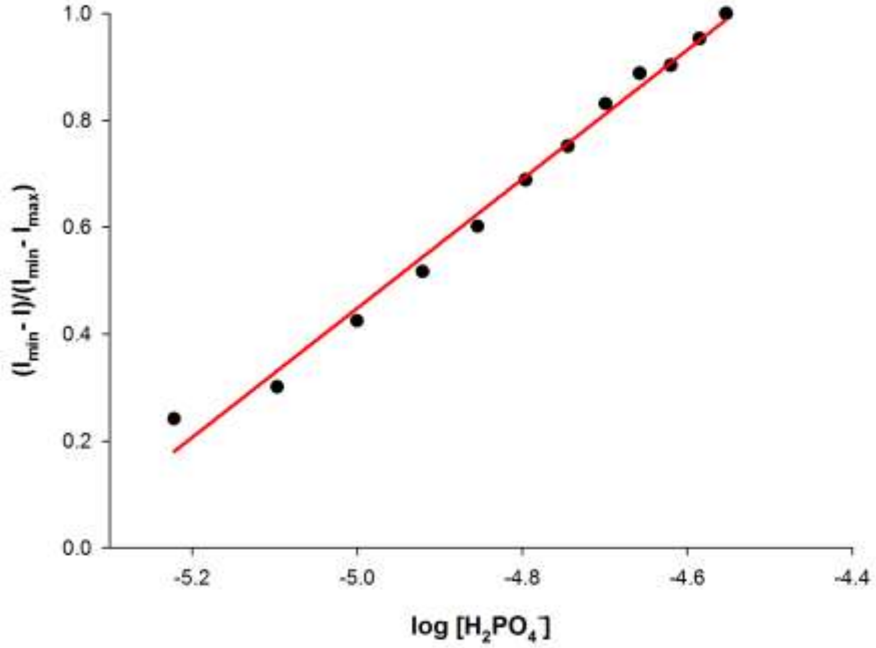

Figure S4. Fluorescence intensity of $\mathbf{5^{2+}{\cdot}2PF_6^-}$ (in CH_3CN) at each concentration of $H_2PO_4^-$ added, normalized between the minimum fluorescence intensity, found at zero equiv of $H_2PO_4^-$; and the maximum fluorescence intensity. The detection limit was found at [$H_2PO_4^-$] = 3.90 $x10^{-6}$M.

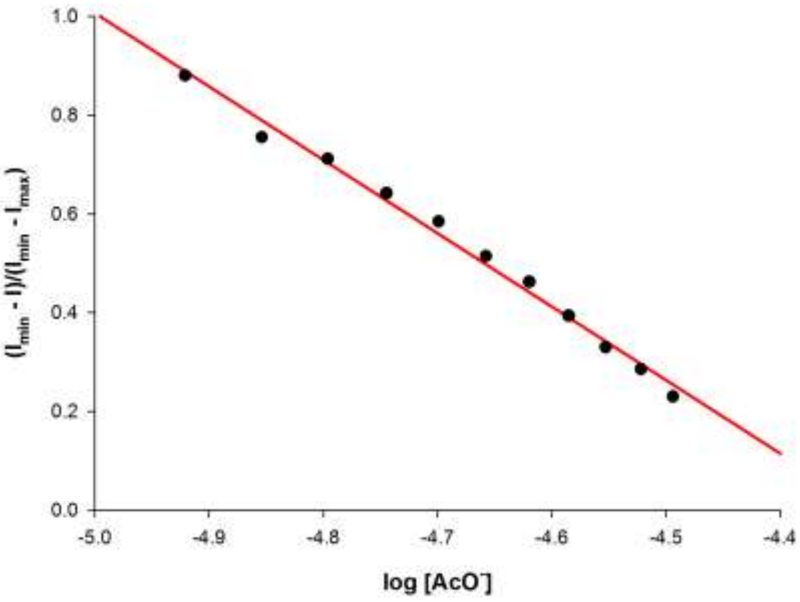

Figure S5. Fluorescence intensity of $\mathbf{5^{2+}{\cdot}2PF_6^-}$ (in CH_3CN) at each concentration of AcO^- added, normalized between the minimum fluorescence intensity, found at zero equiv of AcO^-; and the maximum fluorescence intensity. The detection limit was found at $[AcO^-]= 4.59\ x10^{-5}M$.

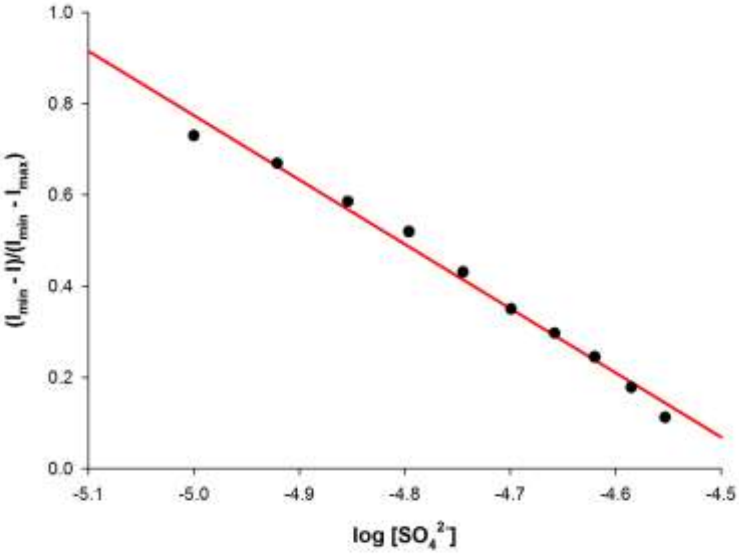

Figure S6. Fluorescence intensity of $\mathbf{5^{2+}{\cdot}2PF_6^-}$ (in CH_3CN) at each concentration of SO_4^{2-} added, normalized between the minimum fluorescence intensity, found at zero equiv of Pb^{2+}; and the maximum fluorescence intensity. The detection limit was found at $[SO_4^{2-}]= 3.57x10^{-5}$ M.

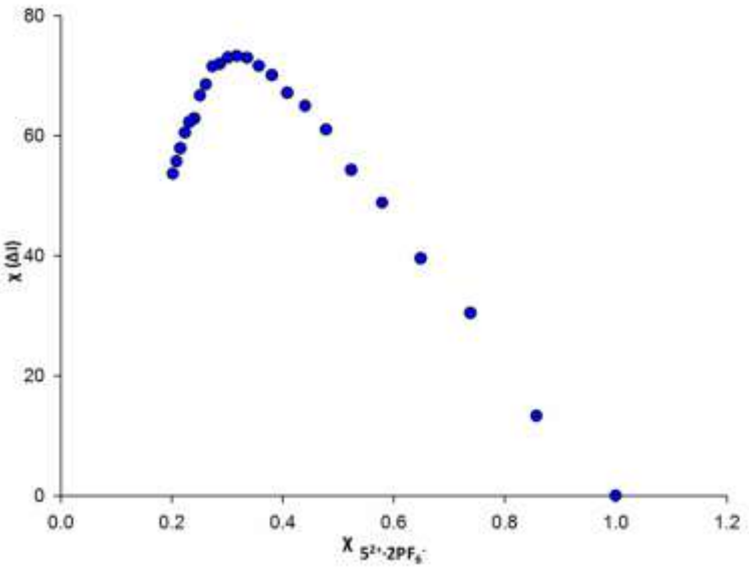

Figure S7. Job plot experiment, using the emission titration data, with a maximum at 0.33 indicating 1:2 stoichiometry for receptor $\mathbf{5^{2+}{\cdot}2PF_6}^-$ and $H_2PO_4^-$ anions.

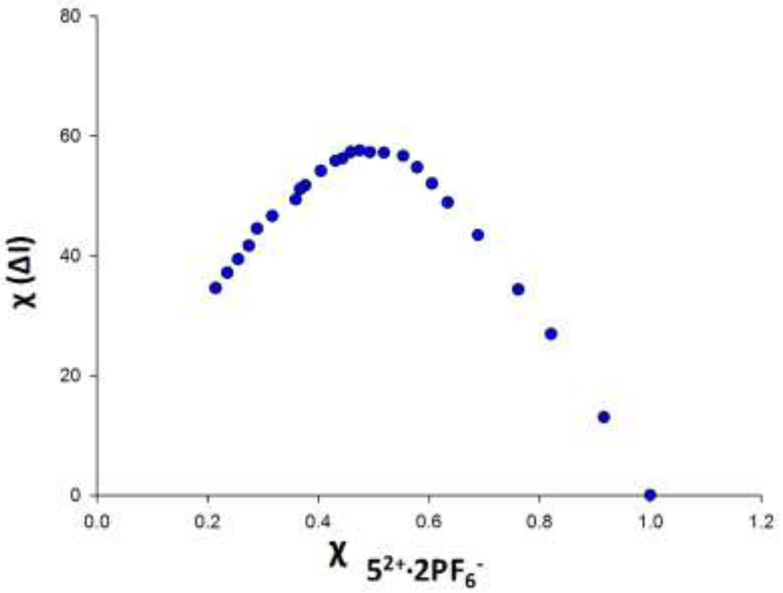

Figure S8. Job plot experiment, using the emission titration data, with a maximum at 0.5 indicating 1:1 stoichiometry for receptor $\mathbf{5^{2+}{\cdot}2PF_6}^-$ and SO_4^{2-} anions.

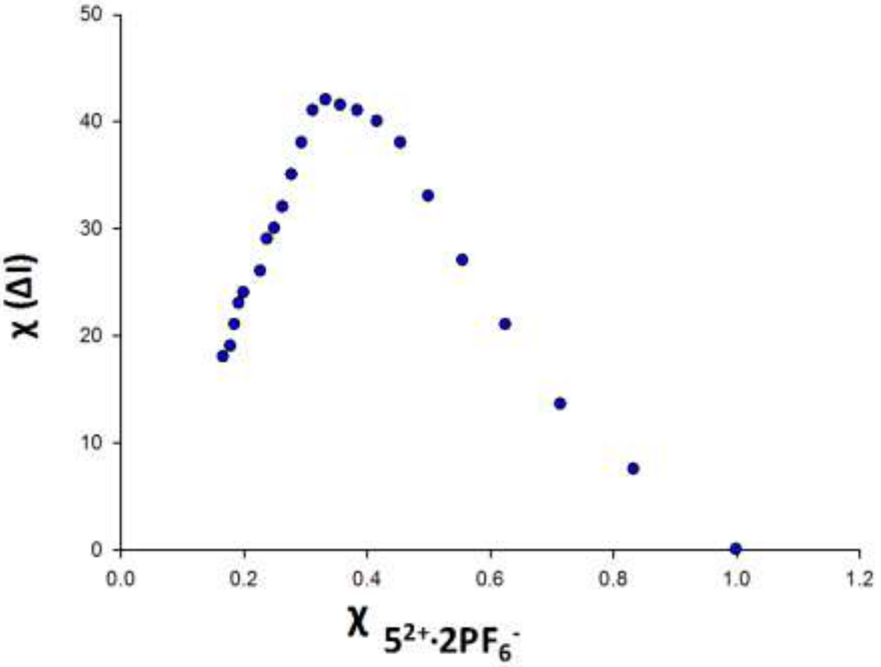

Figure S9. Job plot experiment, using the emission titration data, with a maximum at 0.33 indicating 1:2 stoichiometry for receptor $\mathbf{5^{2+}{\cdot}2PF_6^-}$ and AcO^- anions.

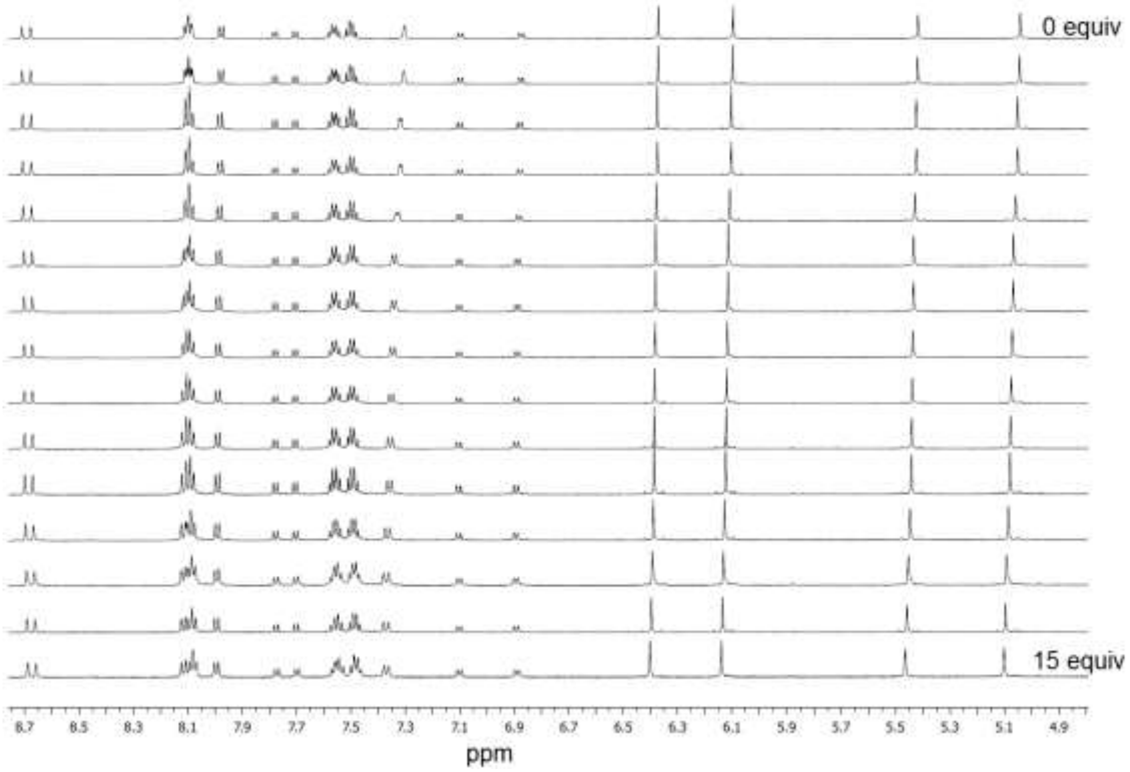

Figure S10. ^{1}H NMR spectral changes observed in the receptor $\mathbf{5^{2+}{\cdot}2PF_6^-}$ in CD_3CN/CD_3OD (8:2, v/v) during the addition of up to 15 equivalents of SO_4^{2-} ions.

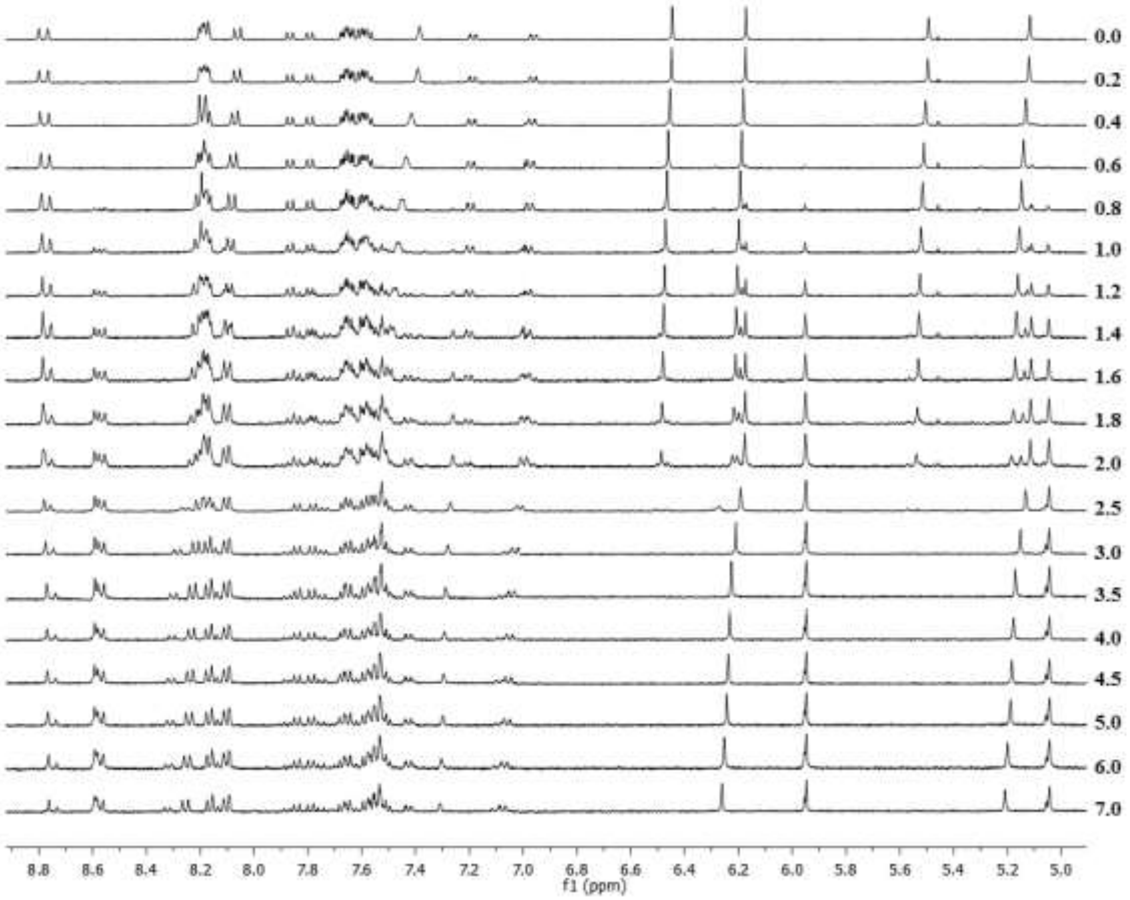

Figure S11. ^{1}H NMR spectral changes observed in the receptor $\mathbf{5^{2+}{\cdot}2PF_6^-}$ in CD_3CN/CD_3OD (8:2, v/v) during the addition of up to 15 equivalents of $HP_2O_7^{3-}$ ions.

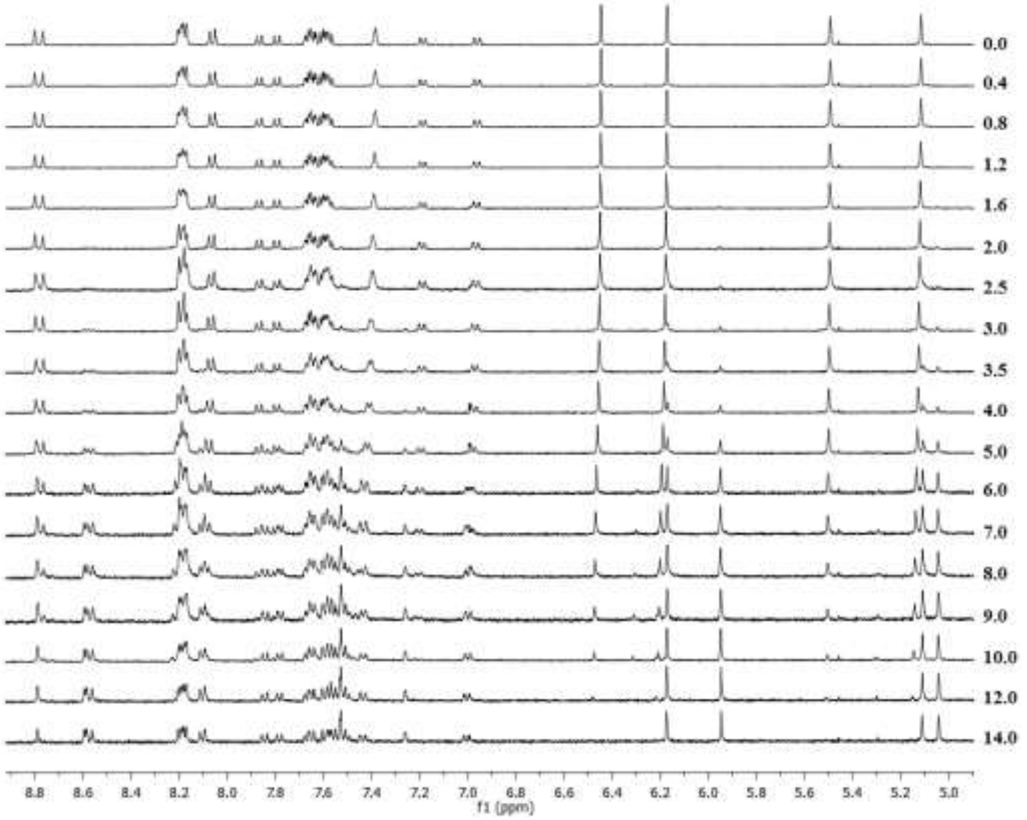

Figure S12. ^{1}H NMR spectral changes observed in the receptor $\mathbf{5^{2+}{\cdot}2PF_6^{-}}$ in CD_3CN/CD_3OD (8:2, v/v) during the addition of up to 15 equivalents of F^- ions.

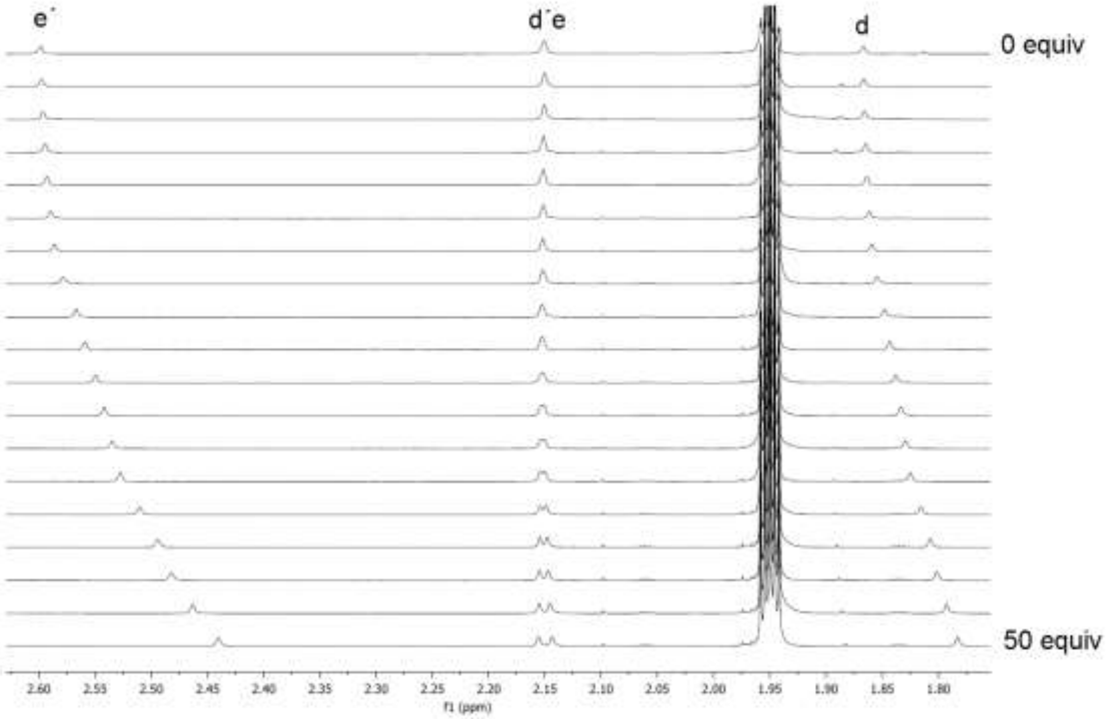

Figure S13. ^{1}H NMR spectral changes observed in the methyl protons of the receptor $\mathbf{5^{2+}{\cdot}2PF_6^{-}}$ in CD_3CN/CD_3OD (8:2, v/v) during the addition of up to 50 equivalents of $H_2PO_4^-$ ions.

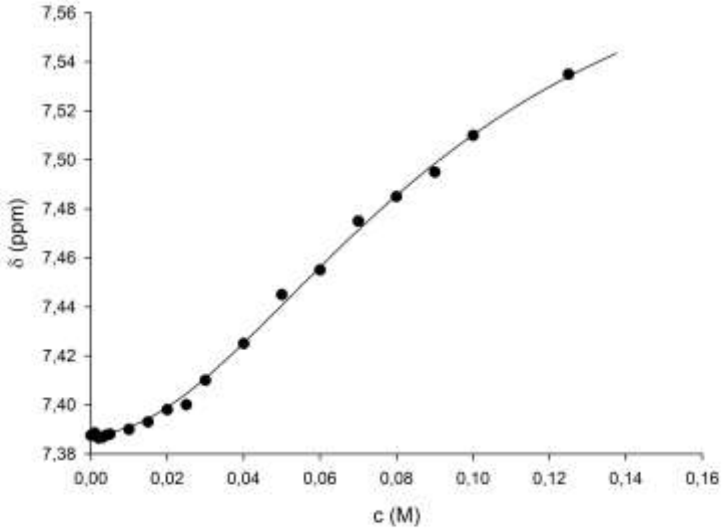

Figure S14. Changes in ^{1}H NMR spectra in the H_a proton of the receptor **5^{2+}·2PF_6^-** upon addition of increasing amounts of $H_2PO_4^-$ anions. Points represent experimental data, continuous lines represent calculate curve.

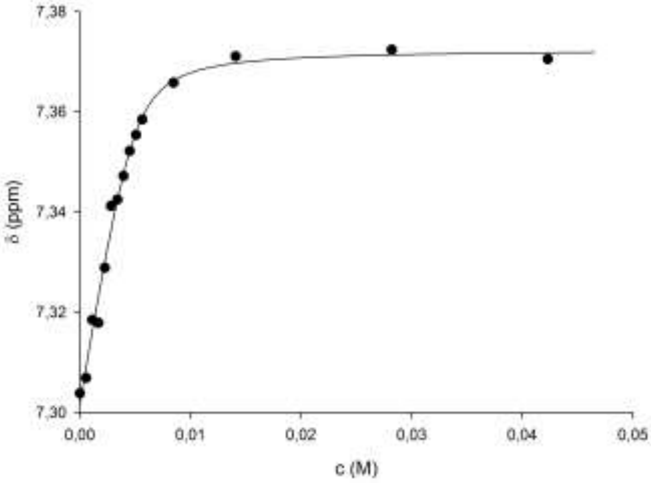

Figure S15. Changes in ^{1}H NMR spectra in the H_a proton of the receptor **5^{2+}·2PF_6^-** upon addition of increasing amounts of SO_4^{2-} anions. Points represent experimental data, continuous lines represent calculate curve.

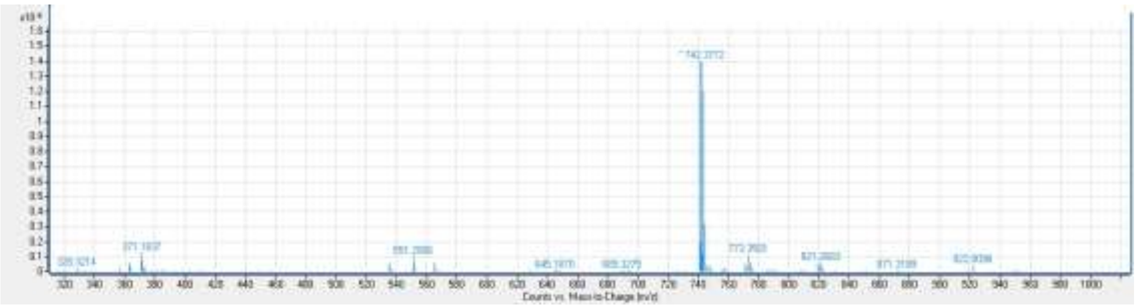

Figure S16. Mass spectrum of the solution of $\mathbf{5^{2+}\cdot 2PF_6^-}$ in CH_3CN after the addition of 3 equivalent of $HP_2O_7^{3-}$ ions, indicating the formation of the imidazolone $\mathbf{6^{+}\cdot PF_6^-}$ $C_{52}H_{45}N_4O^+$ (M^+ = 741.36)
